# Impaired oral health: a required companion of bacterial aspiration pneumonia

**DOI:** 10.3389/fresc.2024.1337920

**Published:** 2024-06-04

**Authors:** John R. Ashford

**Affiliations:** SA Swallowing Services, Nashville, TN, United States

**Keywords:** oral hygiene, pneumonia, aspiration, microbial communities, bacterial aspiration pneumonia, aspiration pneumonia, oral infection control, oral care

## Abstract

Laryngotracheal aspiration has a widely-held reputation as a primary cause of lower respiratory infections, such as pneumonia, and is a major concern of care providers of the seriously ill orelderly frail patient. Laryngeal mechanical inefficiency resulting in aspiration into the lower respiratory tract, by itself, is not the cause of pneumonia. It is but one of several factors that must be present simultaneously for pneumonia to develop. Aspiration of oral and gastric contentsoccurs often in healthy people of all ages and without significant pulmonary consequences. Inthe seriously ill or elderly frail patient, higher concentrations of pathogens in the contents of theaspirate are the primary catalyst for pulmonary infection development if in an immunocompromised lower respiratory system. The oral cavity is a complex and ever changing eco-environment striving to maintain homogeneity among the numerous microbial communities inhabiting its surfaces. Poor maintenance of these surfaces to prevent infection can result inpathogenic changes to these microbial communities and, with subsequent proliferation, can altermicrobial communities in the tracheal and bronchial passages. Higher bacterial pathogen concentrations mixing with oral secretions, or with foods, when aspirated into an immunecompromised lower respiratory complex, may result in bacterial aspiration pneumonia development, or other respiratory or systemic diseases. A large volume of clinical evidence makes it clear that oral cleaning regimens, when used in caring for ill or frail patients in hospitals and long-term care facilities, drastically reduce the incidence of respiratory infection and death. The purpose of this narrative review is to examine oral health as a required causative companionin bacterial aspiration pneumonia development, and the effectiveness of oral infection control inthe prevention of this disease.

## 1 Introduction

Aspiration is one of the contributing causes of many lung diseases, including acute respiratory distress syndrome, aspiration bronchiolitis, aspiration pneumonia, aspiration pneumonitis, exogenous lipoid pneumonia, interstitial fibrosis, bronchiectasis, chronic obstructive pulmonary disease, and asthma (1–4). Bacterial aspiration pneumonia (BAP) (5–7) accounts for 5% to 24% of all types of pneumonia (8), ranks eighth among all causes of death, and is first among infectious diseases causing death (9). Aspiration can be broken down into two components: a pathophysiological event and the aspirate content. Motor/sensory impairment of the larynx is, in and of itself, not the cause of these diseases (10). Larynx closure incompetency due to disease is but an exacerbation of an otherwise normal conveyance of secretions from the oral cavity into the lower respiratory system. The source, content, and volume of the aspirate cause disease.

Efforts to define aspiration pneumonia have been illusory and lacking in completeness and specificity (11). Mandell and Niederman (12) define it as an infection caused by specific microorganisms, while Marik (13) describes it as an infectious process caused by inhalation of oropharyngeal secretions that are colonized by pathogenic bacteria. Ferguson and colleagues (14) contend that use of the term, aspiration pneumonia, is ambiguous and may lead to confusion of the pathogenesis and treatment. They propose using the term, accidental foreign body aspiration, mainly focusing on objects aspirated, such as coins, teeth, nuts, metal objects, and similar materials. Further, the Japanese Respiratory Society (15) adopted a more specific diagnostic definition based on clinical parameters including infiltrates on chest radiographs, suspected or direct confirmation of aspiration, and elevated peripheral white blood cell count. Other factors may include the content and volume of the aspirated material, the frequency of aspiration events, and the host's response to the aspirated material (16).

**Figure 1:**
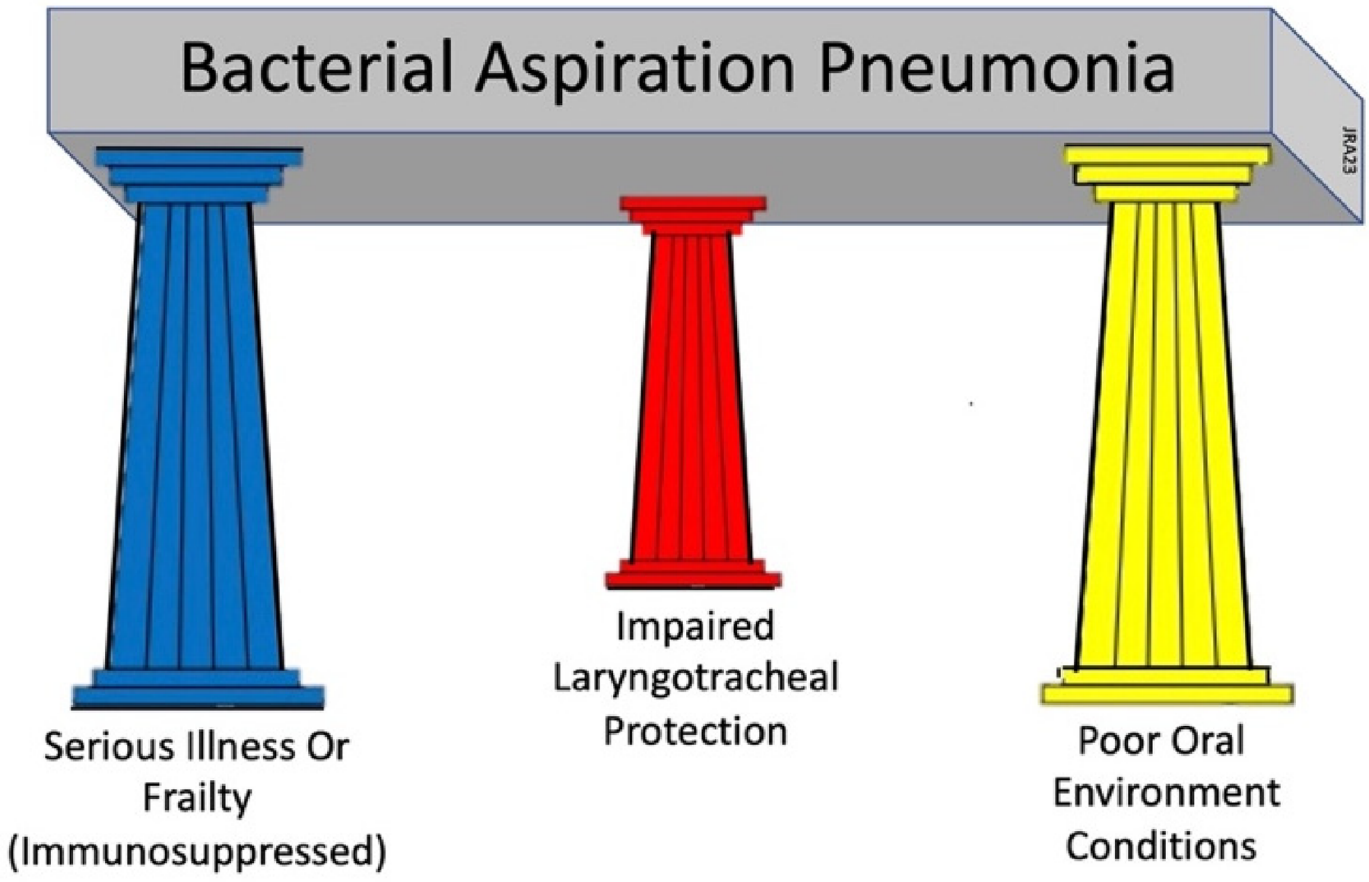
Three pillars of bacterial aspiration pneumonia.

Immune dysregulation, swallowing impairment, recurrent infections, multiple comorbidities, and poor prognosis go well beyond ineffective airway clearing and are common factors found in patients with stroke-associated pneumonia or frailty-associated pneumonia (17). To encompass these factors into a clinically-useable model is challenging. A three-factor model is proposed that prompts equal clinical consideration of the three primary underlying conditions that must be present simultaneously for BAP to develop: (1) the presence of a serious illness or frailty with associated compromised immune functions; (2) the presence of acute oral disease; and (3) the presence of impaired sensorimotor functions of the airway protective mechanism. This model is called the Three Pillars of Bacterial Aspiration Pneumonia (see Figure 1) and defines the three underlying foundational conditions necessary for bacterial aspiration pneumonia to develop. These three factors, or pillars, must be present simultaneously for BAP to develop (18–20). Reducing or eliminating the effects of any one of these three factors through focused treatment significantly reduces the likelihood of BAP developing. The purpose of this narrative review is to examine one of these foundational, but complex factors, the presence of acute oral disease, and its role in BAP development. This review will examine the structures and ecology of the oral cavity, its defenses, its disease contributions to illness, and the effectiveness of oral infection control in the prevention of bacterial-based aspiration pneumonia.

## 2 Normal oral environment—structure and ecology

### 2.1 Oral Mucosa

**Figure 2:**
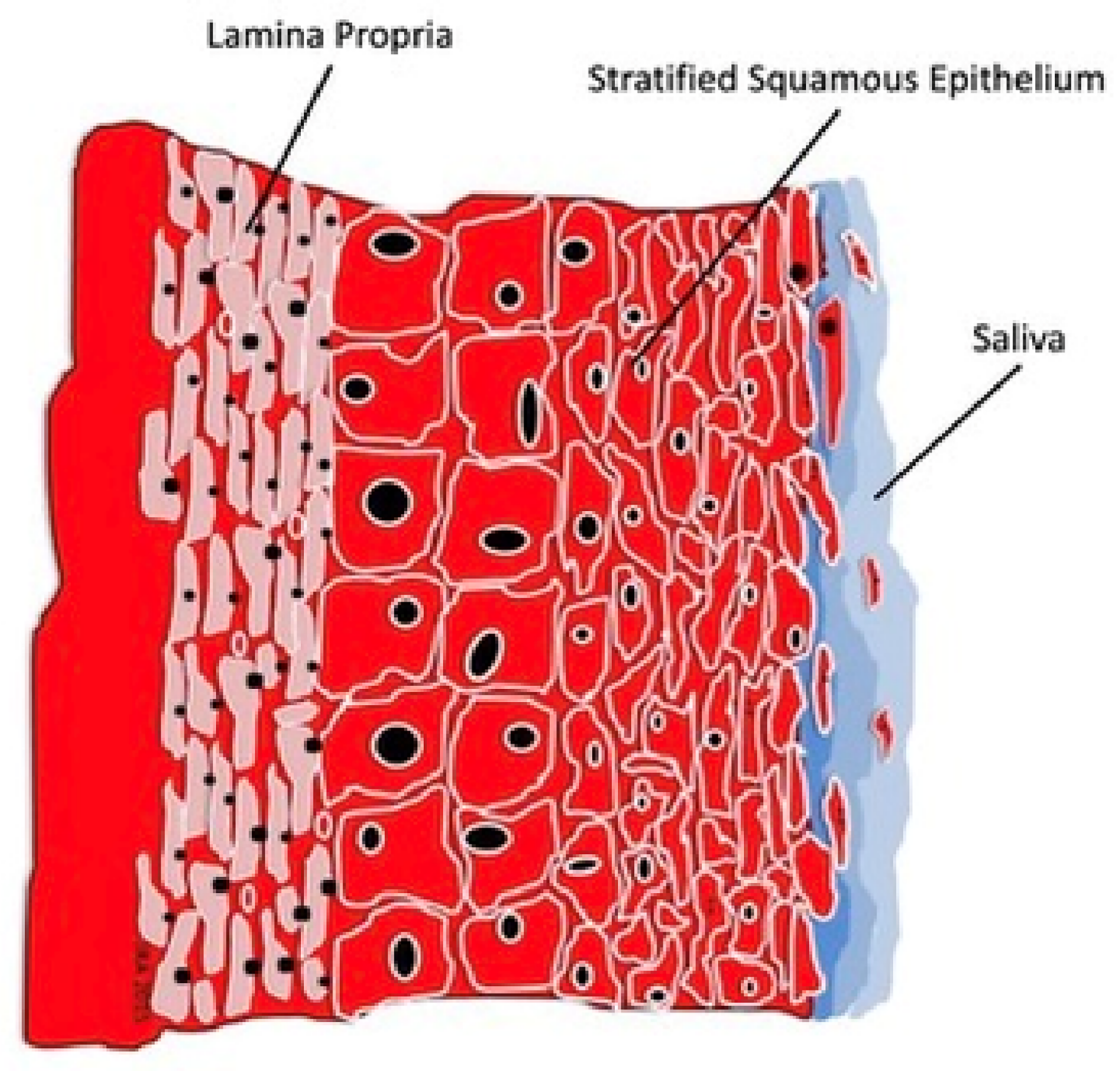
Squamous cell epithelium.

For the oral cavity to remain healthy, the oral mucosa, oral secretions, teeth properties, and the oral microbiota must work in concert to maintain environmental homeostasis. The oral epithelium is an environmentally protective barrier to the tissues deep to its surface (21–23) (see Figure 2). Squamous epithelium is a soft tissue membrane of three-layered divisions: the surface oral epithelium composed of stratified squamous epithelium, an underlying layer of connective tissue or the lamina propria, and the deepest layer composed of dense irregular connective tissue, or the submucosa (22). This epithelial structure, which is comprised of close to 40 structurally overlapping squamous cell layers, cellular cornification, and cell interactions, serves as a protective barrier against external forces. There are roughly 1.540 × 10^7^ superficial or exposed epithelial cells in the mouth (23). Three types of squamous epithelium cover the oral cavity surfaces and differ in histology and function. The lining mucosa is a thin and non-keratinized (elastic or flexible) tissue comprising the surfaces of the cheeks, lips, soft palate, alveolar mucosa, floor of the mouth, and vestibular fornix (21, 22). The masticatory mucosa is a tough epithelium varying in thickness and tightly attaching to hard surfaces such as the hard palate and the base of the teeth. This tight, adhesive mucosa contains keratin and is more resilient and resists deformity by forces generated during mastication (22, 24, 25). With inflammation and tissue breakdown, it becomes a prime site for infection development and for pathogens to colonize. The tongue mucosa, sometimes classified as masticatory mucosa, is a special keratinized squamous epithelium with unique properties including lingual papillae and taste buds (24). The dorsum of the tongue plays an active and crucial role in mastication (22). It's cornified structure, while structurally resistive, allows oral microbes and debris to collect on its surface and provides a location for pathogens to thrive. The surface areas of the normal oral mucosa are sloughed and replaced about every 2.7 h, which prevents bacteria from attaching permanently. With 40 layers of epithelium, 4.5 days are required to completely regenerate the oral mucosa (23).

### 2.2 Oral secretions

The importance of oral secretions, their functions, and contributions to help maintain normal health cannot be understated. Saliva provides the primary watery mechanical and chemical protective covering over all oral surfaces and plays a critical role in oral homeostasis and tissue repair (22, 26, 27). The surfaces of the oral cavity normally remain wet from continuously unstimulated secreted glandular fluid (28). Three pairs of glands–parotid, submandibular, and sublingual–secrete 90% of the saliva (29). The submandibular and sublingual glands provide close to 75% of unstimulated saliva containing mucins. Mucins form a slimy lubricating coating over surfaces to prevent insults to the tissues during eating (30). Clusters of minor salivary glands are dispersed throughout the buccal, labial, distal palatal, and lingual cavity regions and secrete the remaining 10% of the saliva. These glands generally function continuously and secrete mucous with some thinner sero-mucous fluid (28, 31).

Saliva has many functions beyond maintaining oral wetness and these are listed in Table 1. The average saliva flow rate for healthy adults is about 0.3 ml/min with younger adults having a higher flow rate than older adults, and men having higher flow rates than women (23, 26). A healthy person's glands produce roughly 600 ml of saliva per day with the highest flow rates in the afternoon and decreasing during sleep (34). The average oral volume of saliva in the mouth for men before swallowing is 1.1 ml, and after swallowing, 0.8 ml. These volumes are slightly less for women. Thus, with each normal saliva swallowing event, about 0.3 ml is removed from the oral cavity into the airway/digestive structures below (30, 35). Comprised of over 2,000 proteins, peptides, and inorganic compounds (36, 37), some of these proteins provide immune properties such as fibronectin, immunoglobulins, defensins, lactoferrin and glycoproteins (38). Immunoglobulin A (IgA) is an antibody secreted by plasma cells in the salivary glands producing secretory IgA (SIgA). SIgA functions to bind with bacteria preventing them from reaching the oral epithelium (39). While saliva is a poor source of nutrition for bacteria, one ml can contain up to 108 colony forming bacterial units. The constant movement and agitation of saliva works to wash and remove shedding squamous cell surfaces and reduces the potential for bacteria attachment (40).

**Table 1:** Functions of Saliva (32, 33).

Functions of saliva
1. Dilutes substances to stimulate taste receptors.
2. Dilutes harmful sugars.
3. Cleanses oral cavities of bacteria and food residues
4. Lubricates surfaces with mucins to control bacterial and fungal colonization.
5. Buffers or neutralizes acidogenic microorganism that cause tooth decay.
6. Promotes remineralization of tooth enamel.
7. Facilitates the oral preparatory stage of swallowing.
8. Initiates digestion

Dehydration, one of the most common electrolyte disorders among elderly patients, and a primary reason for hospital admission, may directly affect saliva flow (41). As a result, saliva flow rates are reduced, or hyposalivation, increasing saliva protein concentrations and osmolality (42). Fortes and colleagues (43) report that induced exercise dehydration decreased unstimulated saliva flow rate and increased the concentration of SIgA, thus decreasing mucosal immunity protection. Lack of adequate saliva flow movement and agitation may contribute to the development of oral mucositis and increase oropharyngeal colonization with gram-negative bacteria (44). Saliva production and flow decreases are gland-specific and associated with the aging process (45), radiation therapy effects (46), and with the side effects from over 400 medications (47). With aging, low salivary flow rates increase the susceptibility to dental caries due to low buffering capacity of saliva and reduced clearance of oral food debris from tooth surfaces (48, 49). This further increases the risks for oral infection, periodontal disease, and tooth loss (50). Hyposalivation elevates the risks of health complications affecting the older patient's quality of life such as altering dietary practices, nutritional status, taste, speech, and use of dental appliances. Jwabuchi et al. (51) followed over 278 dental patients over the age of 40 for six months to determine the incidence of lower respiratory infections. Sixty percent reported acute respiratory infections over the period with 96 subjects (35%) reporting hyposalivation. Aging, however, does not appear to affect parotid and minor gland saliva flow, which is integral to biofilm formation on tooth enamel, acid neutralization, oral rinsing, and digestion (52). Restoring salivary flow, as a treatment including adequate water intake, may assist in returning the oral cavity to a healthy homeostatic environment reducing pathogenic biofilm formation and the potential for infection development.

Alternative feeding avenues may also impact salivary flow rates and saliva composition. Leibovitz and colleagues (53) examined 23 elderly residents in long-term care facilities using prolonged nasogastric tube feeding (NGT). Compared to a control group, the NGT residents showed alterations in enzyme, elementals, and minerals in saliva composition and a significantly higher rate of oral pathogen colonization. Prolonged nasogastric tube feeding was associated with pathologic oropharynx colonization associated with saliva alterations and related to increased risk for pneumonia from aspiration. Kim and Han (54) examined the salivary flow rates of post-CVA patients and found their flow rates were significantly lower than those of healthy subjects. However, they could not rule out potential effects of medications taken by the stroke group.

Sebaceous glands primarily located close to and surrounding the mouth in the lips, labial, and buccal mucosa secrete small amounts of sebum, a sticky, oily substance. The functions of these oral sebaceous glands have not been clearly determined (22). However, Hoover et al. (55) reported that sebum seals in moisture in deeper cellular levels, promotes lubrication, protects against environmental and infectious insults, and provides immunity functions.

The final oral secretion is gingival crevicular fluid (GCF). It is an exudate released into the gingival sulcus by increased permeability of the capillaries in the gingival tissues in response to inflammation. In the healthy oral environment, these capillaries produce very small amounts of GCF as a serum to flush the gingival sulcus of pathogens and toxic matter and to cushion the tooth against insult (56). Inflammatory immune cells, primarily neutrophils, are present in the dense capillary concentration in the basement membrane and epithelium and help to maintain the health of the gingiva sulcus and fight inflammation. The output flow of GCF maximizes to bathe the area affected by trauma and gingiva inflammation from mastication of course food, dental pocket depth, intracrevicular scraping, scaling, and histamine, and topical application. More recently, GCF analyses have identified protein biomarkers that may reflect early periodontal disease development, as a precursor to potential respiratory infection, and its progression (57, 58).

### 2.3 Teeth

Hard enamel, or carbonated phosphate, composes the structure of teeth and is the only substance that does not regenerate through metabolism (59–61). Heavy concentrations of hair keratins in the enamel resist decay but allow the attachment of biofilms (62). Enamel covers the crown of the tooth and depends on a delicate balancing process of demineralization and remineralization to remain healthy. Remineralization occurs with saliva delivering calcium, phosphate, and fluoride to the surfaces, and from oral cleaning with fluoride toothpastes. Demineralization of the enamel and the underlying dentine results from dietary food acids and lactic acids produced by anaerobic, gram-positive bacteria, such as *Streptococcus mutans*, Streptococcus sobrinum, and lactobacilli (63). Resulting enamel cavities harbor beds of pathogens linked to lower respiratory infections. Cellular or acellular cementum binding covers the root of the tooth, which supports the crown. The root is embedded in the periodontal socket in the alveolar bones of the mandible and maxilla (64, 65). The periodontium is connective tissue consisting of the cementum, the periodontal ligament, alveolar bone and gingival tissue (66). These area locations along the alveolar ridges are primarily where dental disease characteristically develops and becomes the focus of disease prevention.

### 2.4 Oral microbiome

The human oral cavity contains over 700 species of bacteria identified through 16S rRNA sequencing phylogeny (67). Most species are commensal bacteria, or indigenous flora, co-inhabiting on the mucosal and dental surfaces through biofilm development. Chief among the pioneer colonizers are commensal streptococci. This bacteria species is multi-faceted. Some cause enamel demineralization resulting in cavities. Some support other pathogens in periodontitis development. Others interfere with or prevent colonization of tooth surfaces, and still others help modulate the host immune response (68). Immediately after tooth brushing, these pioneer colonizers, or gram-positive bacteria, attach to the tooth surfaces in parallel arrays and extend outward. Secondary and tertiary commensal colonizers attach to these arrays forming biofilms (more later). Most of the oral microbes are commensal, while a few are opportunists with the potential to become pathogenic under certain conditions, or pathobionts (69). By alerting the host immune system to invading oral pathogens, commensal bacteria work to maintain a communal homogeneity among the many species of microbes (70). The total number of bacteria in the healthy mouth at any one time will depend on (1) the number attached to the superficial epithelial cells of the mucosa, (2) the number free floating in saliva, (3) the number attached to epithelial cells floating in saliva, (4) the number in periodontal pockets, and (5) the number attached to teeth (23). As previously stated, there are 1.54 × 10^7^ surface epithelial cells exposed in the mouth. Dawes (23) calculated there are approximately 100 bacteria attached to each epithelial cell, or 1.54 × 10^9^ in total. These flora form into biofilm communities and colonize different sites in the oral cavity (71). Segata and colleagues (72) identified three community groups with distinct bacteria taxonomy: Group 1, buccal mucosa, keratinized gingiva, and hard palate, which harbor a low microbial density; Group 2, saliva, tongue, tonsils, and back wall of oropharynx supporting higher microbial density with the papillated tongue mucosa supporting a highest microbial density; and Group 3, sub-and supra-gingival plaque on tooth surfaces. The non-shedding teeth surfaces accumulate significantly more microbes embedded in dental plaque (73). These attached bacteria can reach more than 10^11^ microorganisms per milligram of dental plaque (74).

Bacteria dispersal within the oral cavity, both actively and passively, determines the overall oral bacteria load present in the cavity at any one time. Active bacterial dispersal occurs through surface erosion, sloughing, and reseeding in spaces on and within the biofilm covering the tooth surfaces. Passive dispersal is from salivary flow forces generated across oral surfaces, surface space competition among bacteria, and dislodging through mechanical forces from teeth occlusion and food mastication (75–77). The number of bacteria floating unattached in saliva accounts for approximately 3.68 × 10^6^ (27.7%) of the total oral bacterial count, while bacteria attached to sloughed squamous cells floating in saliva account for 9.59 × 10^6^ (72.3%), or a total of 13.27 × 10^6^ bacteria suspended in saliva. Thus, most saliva-suspended bacteria are attached to sloughed epithelial cells (23). As noted earlier, most adults swallow approximately 0.3 ml of saliva per swallow event. With a total bacterium count of 13.27 × 10^6^ suspended in saliva, Dawes (23) estimates the bacteria load per swallow of saliva to be 3.619090 × 10^6^ for the orally healthy person, or about 27.3% of the total bacteria load in saliva at the time of the swallow. Bacteria growth doubling in dental biofilms varies from 3 to 14 h depending on the number of layers. Oyetola and colleagues (78) report salivary bacteria loads are significantly higher for subjects with periodontitis compared to those without periodontitis. Using colony counting, they reported the salivary bacteria count was highest among those with poor oral hygiene (1.89 × 10^8^ per ml). A bacteria load of this magnitude in saliva and when aspirated into an immunocompromised lower respiratory system increases the risk of developing bacterial aspiration pneumonia (10, 79, 80).

Berger and colleagues (81) report that environmental factors, diet of individuals, microbial migrations, and genetic factors contribute to the diversity and balance of the oral microbial communities. Opportunistic pathobiontic microbes may turn pathogenic, or foreign pathogens may invade when the host becomes susceptible through immunodeficiency, pathogen infection, and treatment with antibiotics and other drugs (82). Maintaining homogeneity among the commensal bacterial communities is a complex operation involving the host immune system as these microbes' struggle to compete and survive in an ever-changing environment. How a healthy microbiome evolves into a pathobiome is not well understood. Sultan and colleagues (72) describe it as commensal microbes breaching the barrier of commensals becoming pathogenic. This transition results in an overgrowth or imbalance of opportunistic, proinflammatory pathogens disrupting the oral ecosystem balance, or dysbiosis. Oral diseases develop “as a result of a change in the proportion of certain species with greater pathogenic potential within the indigenous flora” (p.4). For an excellent review of the intricacies of the immune system policing the oral environment, see Sultan et al. (73).

Medical science has taught without cited evidence or argument the concept that the lung environment is sterile (83). Cursory understanding of basic human anatomy confirms the airway is constantly open to the outside environment allowing the influx of thousands of particles, bacteria, fungi, and viruses inhaled daily. Under these circumstances, the immune response cannot reasonably maintain a sterile environment. Hilty and colleagues (84) were among the first to challenge the lung sterility belief after culturing samples taken from patients with asthma and COPD and comparing them to normal controls. They identified similar flora in the bronchial tree among all the subjects with asthma, COPD or who were normal. Dickson and associates (83) provide an excellent review of the origins of the notion of lung sterility, and the conceptual errors that have supported this premise. Modern approaches to studying the lower respiratory system microbiome, and without contamination, include collecting the 16S rRNA gene from a bacterial genome and sequencing its single specimens of DNA. Dickson and his group (85), using this method, proposed an adaptive island model of lung biogeography. In the healthy person, the ecosystem is a constant and dynamic migration of microbes via microaspiration from the nasopharynx and oropharynx into the lower respiratory system. This migration supports commensal microbe communities in the lower respiratory system like those found in the oral cavity. In a later paper, Dickson and associates (86) reported the greatest community densities are located at the carina and proximal bronchus intermedius, which coincides with gravity-associated microaspiration flow along the right bronchus. The environmental balance of these lower respiratory microbial populations and their densities are maintained through communal immigration, elimination, and reproduction (83). Evidence strongly supports the direct connection of bacterial communities through mouth-lung immigration with the abundance of similar microbes identified in oral and lung specimens, including *Prevotella sp*. and *Veillonella sp* (86). Ecological homeostasis of these similar commensal communities in the mouth and lungs can abruptly change with the onset of serious illness and accompanying immunocompromise. These changes result in highly virulent bacterial biomasses reducing community diversities. Through oropharyngeal migration via microaspiration of these pathogens into the lower respiratory system, commensal bacterial communities already present in the bronchi become dysbiotic (86–90). The most frequently cultured bacteria in patients with aspiration pneumonia and commonly found in the oral cavity are gram-negative rods, such as *Escherichia coli, Klebsiella pneumoniae, Staphylococcus aureus, and Pseudomonas aeruginosa* (12, 14, 91). This pathogen-dominated imbalance promotes inflammation and subsequent development of respiratory infections, such as BAP (86, 87).

### 2.5 Oral biofilm

**Figure 3:**
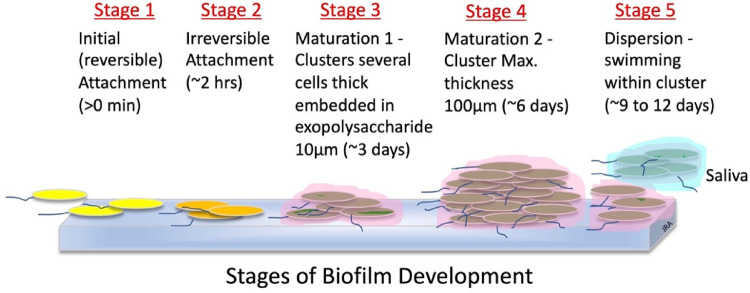
Stages of biofilm development (93).

Biofilms form in natural and industrial systems. Earlier, it was discussed that parallel arrays of layers of slow-growing, commensal bacteria embedded in a gummy glycoprotein and glycolipid (glycocalyx) exudate attach to surfaces, such as the teeth, to form biofilms (69, 91, 92). Sauer and colleagues (93) describe the stages of biofilm development for the bacteria, *Pseudomonas aeruginosa*, and these stages are graphicly depicted in Figure 3. Bacteria encased in biofilm exudate communicate with each other through molecular diffusion called quorum sensing. This signaling ability benefits the bacteria with host colonization, biofilm formation, defense against invader microbes, and adaptation to oral environmental changes. Additionally, quorum-sensing also enables some pathogens to tolerate host defenses and antimicrobial treatments (94).

Dental plaque is an oral biofilm visible around the gingival surfaces of the teeth (95). The teeth, not having the shedding protective properties of the mucosa, are better attachment surfaces for bacteria and dental plaque (79, 90). Saxon, as reported by Rowshani and colleagues (96), states that bacteria begin to recolonize and form new biofilms within three hours of cleaning when adjacent to healthy gingiva. This agrees with Dawes' findings discussed earlier (23). However, if the gingiva becomes inflamed, bacteria recolonization can return within 5 min of cleaning. Bacteria housed and protected in plaque initiate processes responsible for dental caries and periodontal disease discussed earlier. Abdulkareem and associates (77) provide excellent descriptions of the complex processes of biofilm formation and development in the oral cavity.

## 3 Oral care-associated diseases

### 3.1 Dental caries—local disease

Tooth decay is a biofilm-mediated, multifactorial, localized disease and one of the most common preventable diseases across the lifespan (97, 98). As discussed earlier, *Streptococcus mutans* (*S mutans*), a common gram-positive bacterium, and various lactobacilli bacterial species found in the plaque on teeth surfaces cause tooth decay. Person-to-person contact introduces microbes to others, such as a mother or care giver to a new baby. Tooth decay is caused when these pathogens digest sugar forming lactic acids. These acids deplete calcium phosphate in the tooth eroding and penetrating the enamel structure (99, 100). The enamel surface weakens and collapses forming a cavity from demineralization (101, 102). Pathogens may also enter the bloodstream following dental procedures, or from daily dental hygiene practices. Vascular inflammation from these pathogens may result in systemic diseases such as infective endocarditis or may promote tumor metastasis (80). While some studies have not directly linked *S mutans* to aspiration pneumonia, Loesche (103) has linked *S mutans* to tooth decay. In a report by Dye et al. (104), approximately 91% of adults aged 20 to 64 years have dental caries and 27% are untreated. In the 65 years and over, over 96% have dental caries. The number of decayed teeth was a significant predictor (p < 0.01) of pneumonia among 189 elderly long-term care residents in a study by Langmore and associates (10). In a follow-up study of 358 subjects, Terpenning and colleagues (105) identified significant risk factors for BAP to include the number of functional dental units, and the number of decayed teeth, *Streptococcus sobrinus* and *Staphylococcus aureus* in saliva and periodontal disease, and *Porphyromonous gingivalis* in dental plaque.

### 3.2 Periodontal disease—local and system-associated diseases

Periodontal disease is a significant risk factor for BAP (106). This inclusive term is used to describe a group of different biologic conditions causing localized inflammatory disease in the periodontal tissues (74). Assays of oral cavities with periodontal disease, particularly periodontitis, reveal the presence of gram-negative bacteria, such as *Porphyromonas gingivalis, Bacteroides forsythus*, and *Actinobacillus actinomycetemcomitans* (107). This disease results from poor oral health maintenance to remove proinflammatory bacterial-encrusted plaque. These pathogenic bacterial communities release by-products that induce inflammation of the gums and eventual destruction of the bone supporting the teeth (108, 109). For adults 30 years and older, four out of 10 have periodontal disease. Worldwide, 20%–50% of the population has periodontal disease (110, 111).

With the initial onset of periodontal inflammation, the microbial communities become pathobiomes (77, 94, 112, 113). Kinane (108) reports that these communities may be populated by fewer than 10–20 pathogen species and may initiate the onset of periodontal disease within 10 days if the oral environment is poorly cared for. Kinane (108) provides an excellent discussion of the host-based risk factors for periodontal disease progression. Table 2 list some of these factors.

**Table 2:** Host-based risk factors for periodontal disease progression (108).

Periodontal disease risk factors
Aging processes
Poor oral hygiene
Salivary gland dysfunction
Dietary habits
Smoking
Gingival inflammation
Hormonal changes
Socioeconomic status
Race
Medications
Genetic influences
Systemic diseases
Stress, distress and coping behaviors

**Figure 4:**
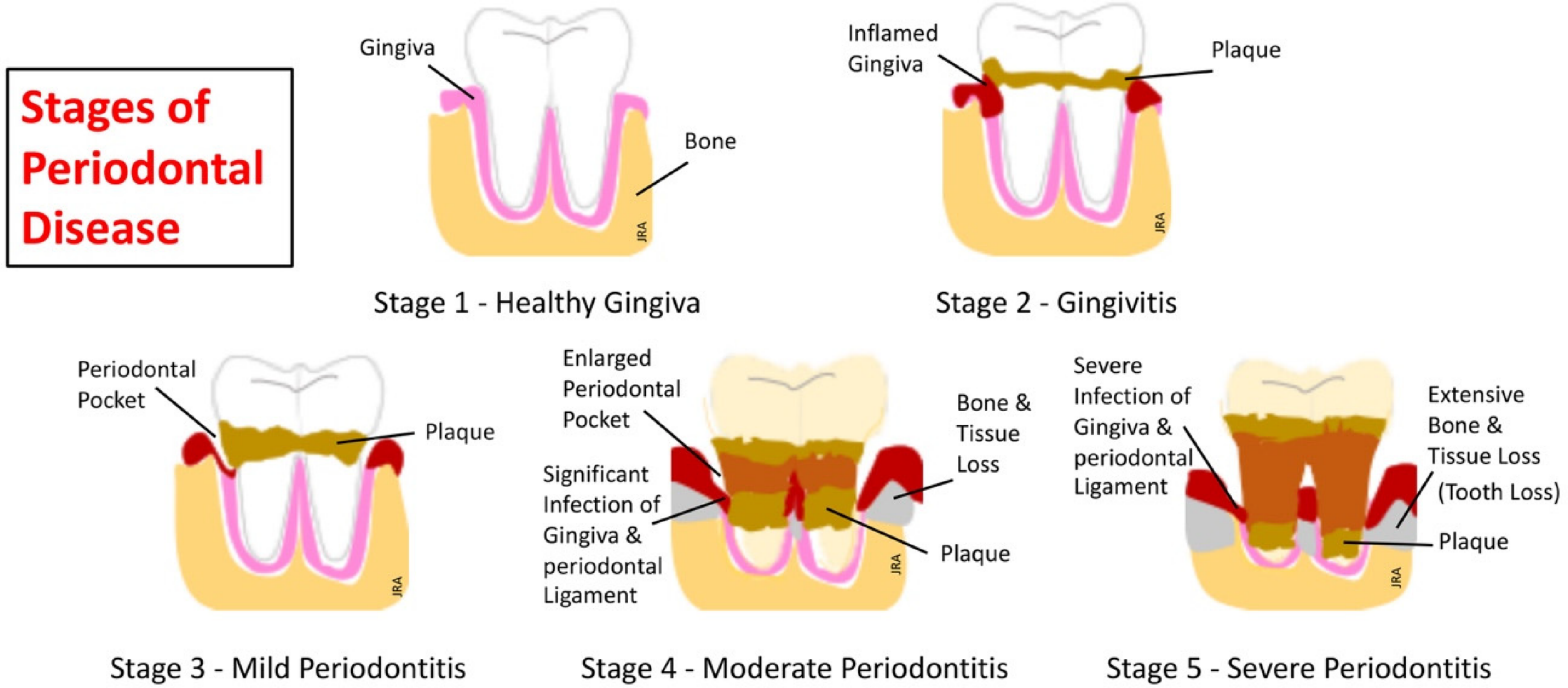
The five stages of periodontal disease (115).

Pathogen-laden biofilms covering the teeth and gingiva evolve and become more attracted to and persist in the inflamed tissue environment. These pathogens are protective and self-sustaining by developing defenses against immune responses and establishing sources of nutrition. Thus, with increasing inflammation of the gingiva, pathogen-laden communities increase their biomasses (114). The most recent model by Van Dyke and his group (115) provides a holistic view of how gingival inflammation is the primary source of plaque-associated periodontal disease. This model describes a 5-stage progression for disease development beginning with healthy gingiva to severe periodontitis and is shown graphically in Figure 4.

Gingivitis, the most common and earliest stage of periodontal disease, develops as a local inflammatory response around the base of the teeth and in the gingival sulcus. This response is limited to the soft gingival epithelium and connective tissue (116). Microbiota assayed at the infected gingiva includes species of gram-negative *Streptococcus, Fusobacterium, Actinomyces, Veillonella,* and *Treponema* (117). If left untreated, gingivitis can potentially progress to periodontitis within 6 months in some individuals (118). Better understanding of this progressive inflammatory process has evolved since the 1960's with evidence placing the focus on bacterial-laden biofilms as a leading factor in periodontal disease development. Subsequently, in a landmark paper, Page and Schroeder (119) describe a four-stage model for the progressive pathogenesis of this disease based upon the body's immune response. This model describes the progressive influx of the innate immune phagocytes, i.e., neutrophils, responding to the initial stage of inflammation and progressing to the adaptive system's antibody-producing plasma cells responding in the advanced stages of the disease. This model, for the first time, provides a foundation for understanding the pathogenesis of periodontal disease. Later models have expanded the Page and Schroder model to help explain the persistence of disease development and to better understand the cellular and molecular mechanisms underlying functions of immune and inflammatory responses (120).

Periodontitis is a low-grade, chronic inflammatory systemic disease that progresses from gingivitis to destroying the periodontium (gingiva, periodontal ligament, and alveolar bone) supporting the teeth in the gingival sulcus (121). A self-perpetuating positive feedback loop forms as the proinflammatory and immune deregulated dysbiotic microbiota in the oral cavity foster destructive inflammation. The resulting inflammation provides a nutritional source for periodontitis-related pathogens, increasing their growth potential (122). The proximity of these oral pathogens to the bloodstream circulating in the gingiva and gingival sulcus can cause bacterial by-products to spread throughout the body, further producing remote acute and chronic inflammation. Numerous studies (123–125) link chronic inflammatory periodontal disease with over 100 systemic diseases. Table 3 lists some of these periodontitis-related systemic diseases.

**Table 3:** Systemic diseases linked to periodontal disease (74, 121, 122, 126–128).

Systemic diseases	
Atherosclerosis	Bacterial pneumonia
Diabetes	Chronic obstructive pulmonary disease
Rheumatoid arthritis	Alzheimer disease
Preeclampsia	Nonalcoholic fatty liver disease
Preterm birth	Colorectal cancer
Inflammatory bowel disease	Chronic kidney disease
Myocardial infarction	Peripheral vascular disease
Stroke	Coronary heart disease
Infective endocarditis	Obesity
Metabolic disorders	Oral cancer
Pancreatic cancer	Esophageal cancer
Emphysema	

Evidence that periodontal disease is a primary causative factor in BAP development is strong (106, 129–135). Cultures from patients with BAP have identified respiratory pathogens including *Porphyromonas gingivalis, Aggregatibacter actinomycetemomitans, Peptostreptococcus, Bacteroides, Prevotella, Fusobacteria, Streptococcus pneumoniae, Hemophilus influenzae, Staphylococcus aureus,* and *Enterobacteriaceae* (133). This connection of pathogens identified in the dysbiotic communities of the oral cavity with those found in the lower respiratory system in patients with pneumonia strongly supports the Three Pillars model advanced earlier. Each pillar is linked by underlying inflammatory processes. Pathogenic biofilms only develop in immune compromised inflammatory conditions in the oral cavity. Pathogens from these biofilms subsequently migrate via saliva-laden microaspiration or food-laden macroaspiration through an inflammatory-induced, mechanically-inefficient larynx and into an immuno-compromised and inflamed lower respiratory system. The result is respiratory disease development, such as BAP.

## 4 Oral hygiene care

### 4.1 Oral hygiene cleaning and rinses

#### 4.1.1 Toothbrushing

**Figure 5:**
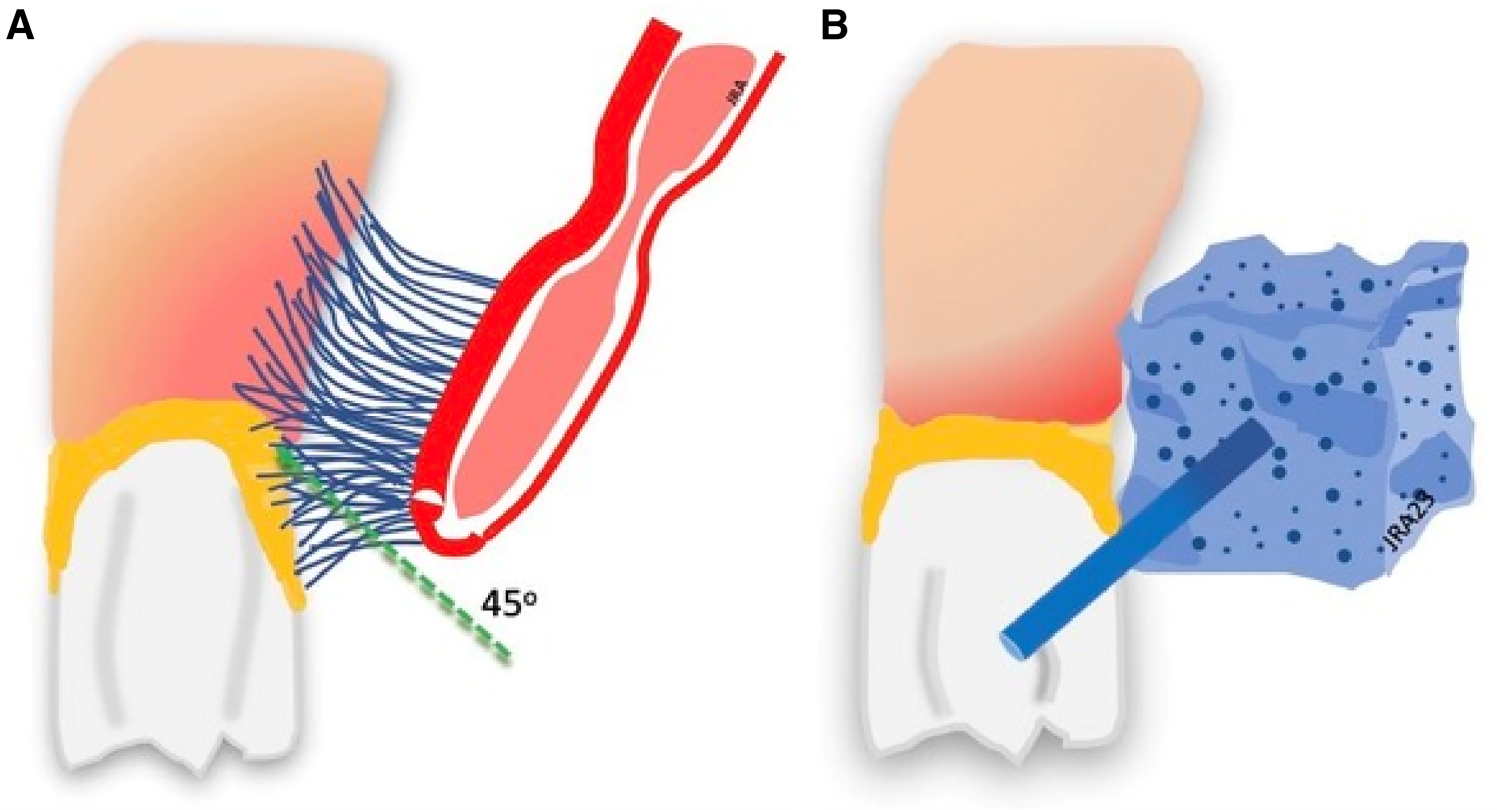
Toothbrushing and toothette cleaning. (**A**) Brushing removes plaque. (**B**) Toothette sponges do not remove plaque.

The toothbrush is the primary tool for cleaning the oral cavity (136). The American Dental Association recommends brushing the teeth twice daily with fluoride toothpaste for two minutes at a 45^o^ angle to clean the crown and the gingiva (137, 138) see Figure 5A.

Most populations do not clean their teeth thoroughly enough to adequately control or prevent dental plaque growth (136). Further, a recognized standard technique does not exist for manually cleaning the teeth and other oral surfaces when caring for people in hospitals or nursing care homes. However, the primary purpose remains the same—removal of bacterial plaque to prevent oral infection–and a bristled brush remains the primary tool. Buglass (139) reports that the primary purposes of oral hygiene care are (1) to maintain a functional and comfortable oral cavity, (2) to enhance self-esteem, and (3) to reduce bacteria activity in the mouth reducing the potential risk of local and systemic infection. Clinical evidence supports the premise that regular oral cleaning reduces oral bacteria and significantly reduces the incidence of BAP (88, 137, 139–144). With ICU ventilator patients, the number of brushings per day may vary from two (145, 146), three (146–149), or four (150). de Lacerda and associates (151), in a prospective, randomized study of 716 ICU patients, report that toothbrushing is associated with a significant reduction in the length of time on the mechanical ventilator. The incidence of ventilator-associated pneumonia (v-BAP) and length of ICU stay were also reduced but without statistical significance. Alhazzani and colleagues (150) report similar findings from a systematic review of six studies of 1,408 patients. Thus, regular toothbrushing with ICU patients may reduce time on ventilation but has smaller effects on reducing the incidence of pneumonia. Nursing care home residents receive toothbrushing less often and less consistently than is recommended by the American Dental Association. Hopcraft's group (152) surveyed 275 Australian long-term care residents and examined the periodontal health of each. Less than one-third of the residents reported brushing their teeth twice or more daily. Less than one-half reported cleaning their teeth only once a day. For residents requiring assistance with oral hygiene from the nursing staff, the frequency and consistency of toothbrushing was very low. Residents with dementia demonstrated poorer oral hygiene than those without dementia, however, the differences were not significant. Overall, periodontal health was extremely poor. Similar findings have also been reported (149, 153, 154).

Hopcraft et al. (152) associated poor oral hygiene in nursing care homes with lack of assistance from staff with brushing, low frequency of brushing, and periodontal disease. Islas-Granillo and colleagues (155) report similar survey findings with adults over 60 years living in long-term care facilities or living in the community. Fifty-three (53.2%) percent of respondents reported brushing their teeth at least once a day. Younger and female participants used oral cleaning aids, such as mouth rinses and dental floss, more often than did older participants. Islas-Granillo et al. further reported that self-dependent residents had better oral hygiene than dependent residents requiring staff assistance. Coleman and Watson (156) report only 16% of residents received oral care from nursing assistants in their observational study. Wagner et al. (157) telemonitored nursing assistants administering oral care over a 100-day period. The average number of days a resident received one brushing per day was 24.45 days. The mean number of days a resident who did not receive oral care at all was 40.38 out of 100. Three months after the researchers discontinued the monitoring program, the residents lost any oral gains made during the monitored trials. Similar results were reported by Gurgel-Juarez et al. (158) for hospital stroke unit patients who received baths 4 times more frequently than oral care. Further, oral care was not documented during the patient's hospitalization in over one-half of the cases.

There have been questions over whether the powered toothbrush is better than the manual toothbrush for removing plaque and preventing gingivitis. Using a Cochrane Database systematic review, Yaacob and associates (159), compared manual and powered toothbrushes in everyday use by people of any age to determine the effectiveness of plaque removal, health of the gingivae, staining, and calculus, among other areas. Reviewing findings of 4,624 participants from 51 trials, they determined that powered toothbrushes provide a significant benefit over manual toothbrushing for reducing plaque and gingivitis with both short-term and long-term use. Several other studies support these findings (160–162). Lavigne and colleagues (163), however, used a single-blind model in a long-term care population to determine if the use of a rotary toothbrush reduced periodontal inflammation compared to usual manual brushing care. After six weeks, they reported no significant differences between the two groups, but both groups showed a reduction in gingiva bleeding. Reduction in gingiva bleeding and inflammation is also reported by Wang (164). Others (165, 166) report modest to no significant differences in dental plaque scores between electric and manual toothbrushes use by long-term care residents. One advantage to using power toothbrushes with the elderly is that they do not require special dexterity as do manual toothbrushes. The powered toothbrush is easier to use with this population and is an excellent alternative to manual toothbrushing (167).

#### 4.1.2 Toothette sponges and swabs

Nursing staffs commonly use lemon glycerin swabs and foam sponges, or toothettes, for oral cleaning instead of soft toothbrushes, particularly with difficult patients or intubated patients. Grap et al. (168) report that sponge toothettes do not work effectively to remove dental plaque. Sponges are not sufficiently abrasive to remove plaque, and cannot penetrate the gingival tissue around the base of the teeth for cleaning see Figure 5B. Huang and colleagues (169) report findings on 282 patients using nasogastric tube feeding and receiving oral care using an oral cleaning sponge only. Those receiving sponge oral hygiene care had a 3.94 times higher rate of pneumonia than those using toothbrush cleanings. Despite evidence that sponges are ineffective for plaque removal, they continue to be a primary tool for oral care (168). Sponges and lemon swabs should be used only to clean the oral mucosal surfaces of excessive mucus collection and other debris from the mouth before toothbrush cleaning or applying liquid antiseptic to the oral surfaces (170).

#### 4.1.3 Rinses

Dentists encourage the practice of swishing liquid in the mouth following eating. Swishing agitation generates pressure around the teeth loosening and removing food particles from tooth crevasses and rinsing sugars from surfaces. Ikeda et al. (171) report that wiping the inside of the mouth with mouth wipes is as effective as rinsing with water and suctioning. That mouth rinses can help control biofilm development leading to halitosis, gingivitis, plaque, and tooth decay is not a recent idea. August Wadsworth (172), a distinguished scholar of Pathology at Columbia University at the first of the twentieth century, recognized that mouth secretions contained virulent bacteria species, both in healthy and diseased individuals. His objective was to destroy these pathogens to prevent pneumonia but discovered they regenerated within hours. Antiseptic mouthwashes were in the early stages of development and he warned that these products should not only destroy the bacteria, but should also be non-abrasive to the oral mucosa, and safe, if swallowed. Early experiments using potassium chlorate, lysol, formaldehyde, hydrogen peroxide, and alcohol showed little to no effectiveness. Of this list, alcohol continues to be used today in some rinses. Mouth washes may be preventative or therapeutic. Preventative oral treatment is the long-term use of a product to control plaque buildup, and therapeutic use is short-termed to assist with oral healing or before and after operative procedures (173). As an antiseptic, the fluid can reach small areas around the teeth acting against the lipids and proteins composing the biofilm, and penetrate to attack bacteria, reducing the bacteria load in the oral cavity (174).

Mouth rinses are classified as cosmetic products and vary in their chemical compositions. The typical mouthwash solutions contain an antiseptic, such as chlorhexidine, cetylpyridinium chloride, methyl salicylate, or providone-iodine. Other ingredients may include water, glycerin, flavoring, artificial coloring, sweeteners, preservatives, emulsifiers, essential oils, and other chemicals (173). Alcohol concentrations in some products may range from 5% to 17% and has been linked to oral mucosa irritation and xerostomia (175, 176). Debate continues over the effectiveness of mouth rinses with different populations. Chlorhexidine is the most commonly used oral antiseptic agent among dentists and physicians in ICU and long-term care facilities (177), but it has not been without its controversy. In the ICU, ventilator-associated pneumonia (VAP) is the leading cause of death with a 50%–76% mortality rate (178). VAP is defined as pneumonia developing more than 48 h after initiating mechanical ventilation (179). Chan et al. (180) systematically reviewed 11 studies totaling 3,242 mechanically-ventilated patients. Four studies (181–183) totaling 1,098 patients found that oral antibiotics did not significantly reduce the incidence of pneumonia, while seven studies (146, 173, 184–189) totaling 2,144 patients reported that oral antiseptics, primarily chlorhexidine, significantly reduced the incidence of VAP. These findings support other studies of chlorhexidine use (190–193). A more recent systematic review of 17 studies by Keykha et al. (194) supports the use of chlorhexidine to reduce the incidence of VAP. However, their review also found chlorhexidine had only a small effect on gram-negative resistant bacteria, which are the most common pathogens causing VAP. Pineda and associates (195) systematically reviewed four studies totaling 1,251 heart surgery and ICU ventilator patients and concluded that the use of the oral antiseptic agent chlorhexidine did not reduce the incidence of nosocomial pneumonia or the rate of mortality. Price and colleagues (196) report selective digestive and oral decontamination were superior to chlorhexidine in preventing death in ICU patients, and, in fact, state that chlorhexidine was associated with a higher rate of mortality in these populations. Further, other studies have reported that chlorhexidine may cause adverse oral mucosa effects including erosive oral lesions, bleeding, ulcerations, and white/yellow plaque (197, 198). Additional evidence now suggests that the effectiveness of chlorhexidine may be pathogen-specific. Fourrier and colleagues (184) followed 228 non-edentulous patients with endotracheal intubation and mechanical ventilation for 28 days. The experimental group received 0.2% chlorhexidine three times daily. Results showed no significant differences in the chlorhexidine group and the placebo group. Chlorhexidine did not eradicate *Pseudomonas aeruginosa, Acinetobacter*, and *Enterobacter* bacterial species from the dental plaque. Some bacteria, such as *Pseudomonas aeruginosa*, form biofilms that protect them from immune invasion, antibiotics, and antiseptic agents, such as chlorhexidine (195). The uncertainty remains and the effectiveness of antiseptic mouthwashes may have to do more with which specific pathogen species are causing the pneumonia and which antiseptics are most effective against those specific pathogens. Studies of mouthwashes using essential oils support their anti-plaque and anti-gingivitis effectiveness (199, 200). Charles et al. (201) followed 108 volunteers for six months. One group rinsed twice daily with a commercial brand of essential oil mouth rinse. A second group rinsed twice daily with chlorhexidine. A control group rinsed with 5% hydroalcohol. At six months, dental exams demonstrated that essential oils mouth rinse and chlorhexidine mouth rinse had comparable anti-plaque and anti-gingivitis effectiveness. Safety concerns remain for children, alcohol addicts, and those with ethanol metabolism deficiencies due to the alcohol content in some of these mouth rinses (200).

### 4.2 Patient oral care programs

#### 4.2.1 Oral assessment procedures

Patient care programs should, ideally, assess the health status of the oral cavity periodically, especially in long-term care facilities. Assessment tools, such as the Minimal Data Set (MDS) or the Resident Assessment Protocol (RAP), are administered at the time of admission, during an annual assessment, or if there has been a significant change in the resident's health status (202). However, these devices may not examine the health of the oral tissues or other oral health-related issues presented by the patient or resident (203). The *Geriatric Oral Health Assessment Index* (GOHAI) helps physicians to identify psychosocial and functional problems associated with oral health issues and to decide if a dental referral is needed. The *Oral Health Impact Profile* (OHIP), developed by Slade and Spencer (204), is an index of physical, social, and psychological descriptors, such as trouble pronouncing words, worse taste, painful aching, self-consciousness, embarrassment, unsatisfying life, etc. The 14-item shorter version is now in use world-wide (205). More recently, Campos and colleagues (206) questioned the validity of the OHIP as a multidimensional measurement. Their study reported that the OHIP-14 works properly as a one-factor model for dentate patients only but not with non-dentate patients. Further, they report that cultural context factors, such as orofacial appearance, or the impact of oral health on life, and age factors could also influence responses, particularly among non-dentate patients. As a measure of the patient's perception of the impact of a given oral condition in their lives, Campos' assessment of the OHIP found it a valid measure. However, self-assessments by older patients or residents are not generally accurate and focus on remaining teeth. In addition, Kayser-Jones et al. (207) report that more than two-thirds of residents have some level of cognitive impairment and cannot report having caries or oral discomfort.

Kayser-Jones and colleagues (207) developed the *Brief Oral Health Status Examination* (BOHSE) to evaluate the oral health of long-term care residents by the nursing staff. It is one of the first screening tools developed to quickly examine ten oral health and function areas (lips, tongue, tissue of the cheek, the roof, and floor of the mouth, gingiva between the teeth or dentures, saliva, condition of natural teeth and dentures, and oral cleanliness). BOHSE uses a 3-point nominal scoring scale (0–2). A summed final score subjectively determines the health status of the oral cavity. A modified version of the BOHSE is the *Oral Health Assessment Tool* (OHAT), a tool designed to simplify the assessment categories and their descriptions. As a staff-administered screening device, it provides practical information to the nursing staff and other care providers about oral hygiene care for functionally dependent and cognitively impaired older adults and helps prevent development of biofilm-related diseases in the oral cavity (201). The OHAT has eight categories and uses the 3-point nominal scoring scale (0-healthy, 1-oral changes, 2-unhealthy) used in the BOHSE. A summed score provides an overall level of oral health. Further, by adding categories for behavioral problems and oral pain, the results of the OHAT may indicate the need for a referral for a dental assessment. Chalmers et al. (208) examined the reliability and validity of the OHAT across 21 nursing care facilities and 455 residents. Amongst the staff, intra-carer agreements were moderate for lips, saliva, oral cleanliness, and referral to a dentist (Kappa = 0.51–0.60), while agreement on all other categories was substantial (Kappa—0.61–0.81). Inter-carer Kappa statistics were similar to the intra-carer agreements. These results support the reliability and validity of the OHAT and its use in nursing care facilities as an oral hygiene screening device. In a retrospective observational study, Maeda and Mori (209) examined 624 hospital-admitted patients over the age of 65 years. The purpose was to determine whether poor oral health could be a predictor of in-hospital mortality within 60 days of the time of hospital admission. The patients were divided into three groups using OHAT scores: (1) Group with OHAT scores of 0; (2) Group with scores of 1 & 2; and (3) Group with scores of >3. Patients with OHAT scores of >3 showed a significantly higher mortality rate (18%) compared to the other two groups. These patients were likely to be older, malnourished, cognitively impaired, and inactive. Primarily used in nursing care facilities, Simpelaere et al. (210) report the OHAT is a very good tool to assess hospital patients when administered competently by the care staff, including nursing, nursing assistants, and speech pathologists.

#### 4.2.2 Oral care as a medical treatment

Oral care, while considered a common and routine hygiene task, is, in fact, a preventative medical treatment for potential oral infection development. Its administration is recognized as a basic nursing duty in hospitals and long-term care facilities in most countries, but may be largely neglected (211, 212). It may either be preventative or responsive. Preventive oral medical treatment is the routine or daily cleaning of the mouth to control bacteria growth and those conditions which may foster the development of disease and illness. Responsive oral medical treatment is purposeful and aggressive oral cleaning for a debilitated person with a serious illness, and to prevent or reduce the risks of secondary illnesses (infections) from developing from oral pathogens. Organized oral care programs reduce the incidence of pneumonia, reduce febrile days, reduce hospital stays, and reduce the incidence of death (213–215). Thus, as a preventative treatment, why isn't oral cleaning a priority in acute care hospitals and long-term care facilities? Salamone and colleagues (211) state that oral health care is an essential duty of nursing care and is a part of a holistic approach including bathing and toileting, or “cares.” While it may be convenient when managing basic patient care duties, nursing should consider separating oral hygiene from this “care package.” Oral hygiene should be reframed as a broader oral infection control procedure and receive the same focused care attention as an infected wound site (217).

Yoon and Steel (217) argue that the use of a holistic approach by caregivers is motivated by social factors and not by potential health consequences related to poor oral hygiene. Lack of proper training and education of the nursing staff in oral health and care is a major concern, but implementation of newly learned care skills is also a factor. Overall, nursing training programs vary in their emphasis on oral care training, and nursing textbooks typically include oral hygiene procedures for those patients unable to manage their own care (218). A survey of recent nursing graduates found that they had a good basic understanding of oral health, but a poorer knowledge and understanding of oral-systemic disease connection and how to screen or examine the oral cavity (219). Dahm et al. (220) report that 1% to 3% of the nursing workforce is trained to provide oral care to older adults with nursing assistants receiving the least training. Unavailable cleaning supplies, uncooperative patients, pressure of other duties, and fear of injury by the patient are reasons given for poor nursing responses related to patient oral hygiene care (211, 221, 222).

Elderly nursing home residents have extensive oral disease and poor oral hygiene (156). In a survey by Wårdh and associates (223), 89% of nursing home staff considered oral health care for residents important; 60% reported brushing teeth was a troublesome activity. Eighty-percent (80%) reported uncooperative residents as a major issue. Similar findings were reported by Palmers and colleagues (224). Facility training programs for continuing education and new staff training in oral care have mixed reviews for effectiveness. Gammack and Pulisetty (225) report that a 30-min staff oral care training program with lecture, demonstrations, and hands-on skill training did not result in significant changes in oral care activities and practices by the staff. Samson and colleagues (226) report that a well-organized program for nursing home residents should emphasize motivating and oral-care training of the staff, use of picture-based oral care cards, distribution of adequate oral care equipment, practical implementation of new routines, and a means to assess outcomes using the mucosal-plaque score index. To test this concept, Samson et al. assessed program effectiveness at three intervals: start of the study, at 3 months and after 6 years. At the start of the program, 36% of the residents had acceptable scores. Six years later, 70% showed acceptable scores. Ildarabadi and associates (227) implemented an 8-week oral care program in a nursing care facility. Improvement was not immediate, and required a minimum of four to eight weeks before improved oral health status were noticeable.

The Mouth Care Without a Battle is a program devised by Zimmerman and colleagues (228). It is a pragmatic program provided by nursing home staff emphasizing person-centered support to improve the resident's quality of life and support the well-being of the staff while providing oral hygiene care. The referenced paper provides a thorough and clear description of the program supported by evidence, and goes well-beyond the scope of this review. In a subsequent paper, Zimmerman and colleagues (229) compared the Mouth Care Without a Battle program with standard oral care in fourteen long-term care facilities. The incidence of BAP was reduced during the first year of the program, but was not significantly changed with the special intervention program during the second year. Sustainability of first year improvement could not be maintained despite staff booster training, and ongoing support. For effective implementation and success of oral care programs in long-term care facilities, a program must be well-organized and documented protocols and procedures, must be administered by a full-time care program director, must have constant staff training, must have adequate equipment, must use valid and reliable measure tools, must use visual tools both for the staff and residents, must keep data and these data must be shared with the staff, and it must have the full support of the facility's administration.

Jones and colleagues (230) surveyed intensive care unit (ICU) nurses regarding their priorities in providing oral care. Thirteen and a half percent (13.5%) rated oral care as a low priority, 85.5% reported using a toothbrush daily with patients, 50.5% routinely used chlorhexidine oral wash, and 23.5% of nurses had not received training in oral care. However, in a later study by Sreenivasan et al. (231), a survey of 200 ICU nurses indicated all were aware of focal oral infection theory, 93% knew about potential complications from poor oral care, and 95% performed oral care after every shift change. They reported the main barrier to oral care with ICU patients was mechanical obstruction secondary to oral intubation and oxygen masks.

Routine oral care neglect increases the possibility of oral-related complications with tube-fed or depressed consciousness patients. In the past, risks of potential aspiration pneumonia and decreased survival have been reasons for the use of tube feeding, nasogastric (NG), or gastrostomy (232). The thinking by some caregivers may be that these patients are not taking food and liquid orally, thus oral care is of lesser importance. Koichiro (233) describes how oral functions are suppressed in tube-fed or depressed consciousness patients and the oral environment is not self-cleaned. As a result, mucosal resting saliva mixes with the oral residue to form a sticky paste-like biofilm that adheres to the oral cavity and teeth surfaces. Reduced salivary washing and mucosa replacement do not remove this biofilm from the oral surfaces and form a coating on the tongue. Dysbiosis of the oral flora allows respiratory pathogens to colonize these thick biofilms and is a viable source for pathogenic aspirate. Blumenstein and colleagues (234) report that poor oral hygiene was found in tube-fed patients with an aspiration incidence of 89%. Juan et al. (235) report a pneumonia rate of 31% in a group of continuous tube-fed stroke patients. Alternative feeding avenues do not prevent microaspiration of pathogen-laden saliva and mucous generated in the oral and pharyngeal cavities (236). Luk and Chan (232) state that tube feeding should be a last resort and should not be the rationale to prevent pneumonia. In a retrospective study of 63 patients receiving enteral feeding or restricted oral foods, Maeda and Akagi (237) reported that a formalized oral hygiene care program was effective. They used two groups (control and an oral hygiene program group). The incidence of pneumonia for the tube-fed or restricted oral feeding group receiving formal oral care from the staff was significantly less than in the control group (0.45 vs. 1.20). In addition, oral care in the intervention group reduced febrile days, reduced administration of antibiotics, and reduced the number of blood tests and radiographic studies taken.

#### 4.2.3 Professional oral care

Professional oral care provisions in health care facilities vary worldwide. Few hospitals in the United States provide inpatient or outpatient dental services, with dentistry provided through private dental practices, which is the universal model. An exception is the inpatient and outpatient dental services provided to military veterans by the U.S. Department of Veterans Affairs hospitals. In many countries, dental services are funded through private pay or some form of private or government-supported insurance. While long-term care facilities in the U.S. are federally mandated to assess the oral health of their residents, few facilities comply. The lack of dentist availability and costs prevent long-term care facilities from providing onsite dental services (238). Use of dental hygienists has increased and has shown to be effective in preventing respiratory infections with nursing home residents (239). Other facilities have utilized dental hygienists as staff coaches to implement and guide oral care programs, such as the Mouth Care without a Battle (240). More recently, several countries, such as the United Kingdom, Australia, and New Zealand, have created a new specialty, Oral Health Therapy (OHT). This specialty's scope of practice includes oral health assessment, examination, diagnosis and treatment planning, prevention, minimal intervention and health promotion as well as nonsurgical treatment of periodontal disease and dental caries. In these countries, the OHT duties include some of the same duties of dental hygienists and dental therapists (241). OHTs have become valuable resources long-term care facilities, particularly with the frail elderly. However, many of the same barriers exist as with dental hygienists including lack of opportunity, adequate education and training, poor pay, and having adequate equipment (242).

Weekly professional, mechanical cleaning vs. daily antiseptic disinfecting decreases or eliminates oropharyngeal bacteria in the dependent elderly (243). Adachi and colleagues (244) followed 141 elderly nursing home residents for two years. Those receiving professional oral care weekly had significantly reduced fevers and fatal BAP when compared to a control group of residents receiving routine daily care. Similarly, Ishikawa et al. (243) followed three cohorts receiving staggered routines of professional care for five months. Results showed that bacteria counts were significantly lower in all three groups following professional care for 5- and 3-month periods. At 3 months, group 3 began receiving professional care and with significant reductions in Streptococci and Candida. Febrile days increased for group one (not significant) and group two (significant) but decreased in group three. The effects of the cold and flu season during the study influenced the latter findings. Pneumonia developed in 8 residents in group one and no cases in group two or three during the experimental period. In a study by Sjögren and colleagues (245), oral care significantly reduced mortality when provided by dental personnel compared with the care administered by the nursing staff. Further, the incidence of mortality did not significantly change with nursing staff administered oral care. Finally, in a seminal study, Yoneyama et al. (246) randomly assigned 417 nursing home residents to one of two groups: oral care group and no-oral care group. The no-care group received routine oral care, while the care group received daily assistance from caregivers and nurses, and dentists or dental hygienists visited weekly to provide professional care. This organized oral care program significantly reduced the occurrence of pneumonia, febrile days, and death in this population. The inclusion of professional oral care successfully reduces the incidence of pneumonia by as much as 40% among the elderly in the care group (213). While professional dental services are not widely available in many countries, in those countries where it is provided the incidence of pneumonia and death in residents in long-term care facilities is reduced.

## 5 Discussion

Pathogenic flora residing in the oral cavity cause local and systemic diseases including periodontal disease and bacterial aspiration pneumonia. This narrative review examines this complex ecosystem and how it changes with aging and impaired health status. These changes can potentially trigger a cascade of microbiological events that result in local and other systemic diseases. This review does not include aspiration of gastric contents, or aspiration pneumonitis. The intended focus is to examine the complexity of the oral cavity environment, its microbiome, its pathological changes that lead to development of BAP, and the effectiveness of oral care intervention in the prevention of BAP.

The term, “bacterial aspiration pneumonia,” is used in this review as it has appeared in many prior publications (221, 247–250) in place of the commonly used term, “aspiration pneumonia.” This particular terminology specifically emphasizes that bacteria is the required component when aspiration from the oropharynx results in pneumonia. Further, this term distinguishes bacteria aspiration pneumonia from other terms often used to imply pulmonary infection, such as “aspiration pneumonitis,” “post-obstructive pneumonia,” “community-acquired pneumonia,” “ventilator-associated pneumonia,” or “hospital-acquired pneumonia.” Many of these terms rely on descriptors of population or environment locations, implied equipment-associated causes, or other conditions rather than the underlying bacterial pathogen cause. Some designators are more specific and stipulate the type of infections causing the pneumonia, such as Staphylococcus pneumonia, Staphylococcus aureus pneumonia, or Klebsiella pneumonia. Adopting the use of this microbe-based terminology clinically helps differentiate it from other terms used for aspiration pneumonia and provides clarity of meaning for care providers much the same as “viral pneumonia” differentiates itself from bacteria-based pneumonias.

As discussed, for pneumonia to develop, the environment of the oral cavity must be dysbiotic with impaired airway protection and compromised lower respiratory immunity. The Three Pillars of Bacterial Aspiration Pneumonia model brings focus to these three primary factors. This model identifies serious illness (Pillar I), poor oral health (Pillar II), and laryngotracheal impairment (Pillar III) as the primary components that must be present together for bacterial pneumonia to develop (18–20). If this model is inclusive of all factors needed for BAP development, it may differ from other models because it places the health status of the oral cavity and it's aspirate as key factors, and holding equal or higher importance than the biomechanical inefficiency of the larynx resulting in aspiration. Many papers have presented excellent definitions of aspiration pneumonia, and the “Sekizawa Definition” provided by the Japanese Respiratory Society in 2009 (15) is exemplary for its guidance in pneumonia diagnoses. But, like many descriptors, it focuses on the identification and diagnosis of pneumonia and not on its source, the oral cavity. To include the importance and complexity of an unhealthy oral cavity environment to pneumonia development helps to better frame the complexity of pneumonia, particularly in clinical assessments and interventions. Bacteria aspiration pneumonia may be defined as the result of biomechanical and sensory inefficiency or impairment of the protective laryngeal valving mechanism allowing virulent pathogens originating in the oral cavity to enter an immunocompromised lower respiratory system and infecting the lung parenchyma.

To augment the human immune system efforts to control pathobiome development, oral hygiene care works to control and reduce biofilm accumulations of pathogen colonies on oral surfaces. Toothbrushing regularly is the number one method in reducing biofilms from the surfaces of teeth, the primary sites of bacterial attachment. Controversy over the use of chlorhexidine has not been definitively resolved. While it appears in many studies to effectively reduce bacterial load and prevent BAP development, questions remain over its effective dose, potential side effects, population-specific outcomes, and its impact on motality. The validity of chlorhexidine use in specific populations remains unclear and further double-blind studies are needed (251).

Oral hygiene care seems simple enough. Pressing and scrubbing a bristle brush against the surfaces of the teeth. However, when caring for others, this seemingly simple task may often be overlooked, neglected, or too challenging for caregivers. Nursing assistants or aides are given the task with little medical knowledge of the oral cavity or how to properly provide oral cleaning. Even for the educated caregiver, patient, or nursing home resident, oral cleaning processes may be difficult and time-consuming. Often, proper equipment, products, and assistance are not available. And, providing care to disruptive patients or residents becomes even more challenging for the staff. Successful programs are presented and referenced in this paper and have shown ongoing staff education, leadership, and teamwork provide the best results for the patients and residents. Oral hygiene prevents diseases and facilitates a better quality of life for patients. This fact should elevate its importance in all care facilities-hospitals and long-term care facilities-toward disease prevention. Oral care or oral hygiene tasks should be recognized and elevated to the status of oral infection control.

While this review can only be considered cursory, much more is known about the importance of oral health through a rich and vast repository of studies, data and findings. The evidence that oral pathogens cause systemic diseases is not new. Wadsworth (172) told us so over a hundred years ago. And, evidence supporting oral cleaning as the best intervention to help prevent these diseases is not new and very plentiful. The questions then are (1) why is oral health and oral cleaning not a primary focus in healthcare and disease prevention, and (2) why is preventative and restorative oral health care not considered on an equal basis as other medical care for payment support, such as insurance and governments-supported health care plans?

## 6 Conclusions

Oral hygiene care, if utilized as a medical treatment, prevents systemic disease, particularly bacterial aspiration pneumonia. While acknowledged as a patient-care procedure, oral cleaning is overlooked or neglected in hospitals and nursing care facilities. Poor staff training, lack of supplies, and unsupportive administrators are the primary obstacles in providing this preventative care to hospital patients and nursing home residents. Concerted and well-organized preventative oral care program reduce the incidence of pneumonia and death and improve patient quality of life.

## References

[1] Lee AS Ryu JH. Aspiration pneumonia and related syndromes. In: Mayo Clin Proc.; 2018 Jun 1; Elsevier (2018). Vol. 93, No. 6, pp. 752–62. 10.1016/j.mayocp.2018.03.011 29730088

[2] Prather AD Smith TR Poletto DM Tavora F Chung JH Nallamshetty L Aspiration-related lung diseases. J Thorac Imaging. (2014) 29(5):304–9. 10.1097/RTI.0000000000000092 24911122

[3] Hu X Lee JS Pianosi PT Ryu JH. Aspiration-related pulmonary syndromes. Chest. (2015) 147(3):815–23. 10.1378/chest.14-1049 25732447

[4] Lee AS Lee JS He Z Ryu JH. Reflux-aspiration in chronic lung disease. Ann Am Thorac Soc. (2020) 17(2):155–64. 10.1513/AnnalsATS.201906-427CME 31697575

[5] Pennza PT. Aspiration pneumonia, necrotizing pneumonia, and lung abscess. Emerg Med Clin North Am. (1989) 7(2):279–307. 10.1016/S0733-8627(20)30337-0 2653801

[6] Tortuyaux R Voisin B Cordonnier C Nseir S. Could polymerase chain reaction–based methods differentiate pneumonitis from bacterial aspiration pneumonia? Crit Care Med. (2018) 46(1):e96–7. 10.1097/CCM.0000000000002744 29252957

[7] Howard J Reinero CR Almond G Vientos-Plotts A Cohn LA Grobman M. Bacterial infection in dogs with aspiration pneumonia at 2 tertiary referral practices. J Vet Intern Med. (2021 ) 35(6):2763–71. 10.1111/jvim.16310 34751462 PMC8692172

[8] Hirooka N Nakayama T Kobayashi T Nakamoto H. Predictive value of the pneumonia severity score on mortality due to aspiration pneumonia. Clin Med Res. (2021) 19(2):47–53. 10.3121/cmr.2020.1560 33547167 PMC8231691

[9] Regunath H Oba Y. Community-acquired pneumonia. In: InStatPearls. Treasure Island, FL: StatPearls Publishing (2021). p. 1–8. 28613500

[10] Langmore SE Terpenning MS Schork A Chen Y Murray JT Lopatin D Predictors of aspiration pneumonia: how important is dysphagia? Dysphagia. (1998) 13:69–81. 10.1007/PL00009559 9513300

[11] Teramoto S. The current definition, epidemiology, animal models and a novel therapeutic strategy for aspiration pneumonia. Respir Investig. (2022) 60(1):45–55. 10.1016/j.resinv.2021.09.012 34782300

[12] Mandell LA Niederman MS. Aspiration pneumonia. N Eng J Med. (2019) 380(7):651–63. 10.1056/NEJMra1714562 30763196

[13] Marik PE. Aspiration pneumonitis and aspiration pneumonia. N Eng J Med. (2001) 344(9):665–71. 10.1056/NEJM200103013440908 11228282

[14] Ferguson J Ravert B Gailey M. Aspiration:/asp’rāSH () n: noun: an ambiguous term used for a diagnosis of uncertainty. Clin Pulm Med. (2018) 25(5):177–83. 10.1097/CPM.0000000000000277

[15] Japanese Respiratory Society. Aspiration pneumonia. Respirology. (2009) 14(Suppl 2):S59–64. 10.1111/j.1440-1843.2009.01578.x 19857224

[16] Lee JS Collard HR Raghu G Sweet MP Hays SR Campos GM Does chronic microaspiration cause idiopathic pulmonary fibrosis? Am J Med. (2010) 123(4):304–11. 10.1016/j.amjmed.2009.07.033 20362747 PMC2851633

[17] Smithard DG Yoshimatsu Y. Pneumonia, aspiration pneumonia, or frailty-associated pneumonia? Geriatrics. (2022) 7(5):115. 10.3390/geriatrics7050115 36286218 PMC9602119

[18] Bartlett JG Gorbach SL. The triple threat of aspiration pneumonia. Chest. (1975) 68(4):560–6. 10.1378/chest.68.4.560 1175415

[19] Niederman MS. Nosocomial pneumonia in the elderly patient: chronic care facility and hospital considerations. ClinChest Med. (1993) 14(3):479–90. 8222564

[20] Ortega O Parra C Zarcero S Nart J Sakwinska O Clavé P. Oral health in older patients with oropharyngeal dysphagia. Age Ageing. (2014) 43(1):132–7. 10.1093/ageing/aft164 24190874

[21] Groeger S Meyle J. Oral mucosal epithelial cells. Front Immunol. (2019) 10:208. 10.3389/fimmu.2019.00208 30837987 PMC6383680

[22] Brizuela M Winters R. Histology, Oral Mucosa. Treasure Island, FL: StatPearls Publishing (2022). Available online at: https://pubmed.ncbi.nlm.nib.gov/34283481/34283481

[23] Dawes C. Estimates, from salivary analyses, of the turnover time of the oral mucosal epithelium in humans and the number of bacteria in an edentulous mouth. Arch Oral Bio. (2003) 48(5):329–36. 10.1016/S0003-9969(03)00014-1 12711376

[24] Chen J Ahmad R Li W Swain M Li Q. Biomechanics of oral mucosa. J R Soc Interface. (2015) 12(109):20150325. 10.1098/rsif.2015.0325 26224566 PMC4535403

[25] Schroeder HE Listgarten MA. The gingival tissues: the architecture of periodontal protection. Periodontol 2000. (1997) 13(1):91–120. 10.1111/j.1600-0757.1997.tb00097.x 9567925

[26] FenolI-Palomares C Muñoz-Montagud JV Sanchiz V Herreros B Hernández V Mínguez M Unstimulated salivary flow rate, pH and buffer capacity of saliva in healthy volunteers. Rev Esp Enferm Dig. (2004) 96(11):773–83. 10.4321/s1130-01082004001100005 15584851

[27] Pedersen AM Sørensen CE Proctor GB Carpenter GH. Salivary functions in mastication, taste and textural perception, swallowing and initial digestion. Oral Dis. (2018) 24(8):1399–416. 10.1111/odi.12867 29645367

[28] Eliasson L Carlén A. An update on minor salivary gland secretions. Eur J Oral Sci. (2010) 118(5 ):435–42. 10.1111/j.1600-0722.2010.00766.x 20831576

[29] Saitou M Gaylord EA Xu E May AJ Neznanova L Nathan S Functional specialization of human salivary glands and origins of proteins intrinsic to human saliva. Cell Rep. (2020) 33(7):1–15. 10.1016/j.celrep.2020.108402 PMC7703872 33207190

[30] Fatima S Rehman A Shah K Kamran M Mashal S Rustam S Composition and function of saliva: a review. World J Pharm Pharm Sci. (2020) 9(6):1552–67. 10.20959/wjpps20206-16334

[31] Iorgulescu G. Saliva between normal and pathological. Important factors in determining systemic and oral health. J Med Life. (2009) 2(3):303. 20112475 PMC5052503

[32] Tabak LA. In defense of the oral cavity: structure, biosynthesis, and function of salivary mucins. Annu Rev Physiol. (1995) 57(1):547–64. 10.1146/annurev.ph.57.030195.002555 7778877

[33] de Almeida PD Gregio AM Machado MA De Lima AA Azevedo LR. Saliva composition and functions: a comprehensive review. J Contemp Dent Pract. (2008) 9(3):72–80. 10.5005/jcdp-9-3-72 18335122

[34] Vila T Rizk AM Sultan AS Jabra-Rizk MA. The power of saliva: antimicrobial and beyond. PLoS Pathog. (2019) 15(11):e1008058. 10.1371/journal.ppat.1008058 31725797 PMC6855406

[35] Lagerlof F Dawes C. The volume of saliva in the mouth before and after swallowing. J Dent Res. (1984) 63(5):618–21. 10.1177/00220345840630050201 6584462

[36] Denny P Hagen FK Hardt M Liao L Yan W Arellanno M The proteomes of human parotid and submandibular/sublingual gland salivas collected as the ductal secretions. J Proteome Res. (2008) 7(5):1994–2006. 10.1021/pr700764j 18361515 PMC2839126

[37] Heo SM Choi KS Kazim LA Reddy MS Haase EM Scannapieco FA Host defense proteins derived from human saliva bind to Staphylococcus aureus. Infect Immun. (2013) 81(4):1364–73. 10.1128/IAI.00825-12 23403559 PMC3639616

[38] Fábián TK Hermann P Beck A Fejérdy P Fábián G. Salivary defense proteins: their network and role in innate and acquired oral immunity. Int J Mol Sci. (2012) 13(4):4295–320. 10.3390/ijms13044295 22605979 PMC3344215

[39] Matsuzaki K Sugimoto N Islam R Hossain ME Sumiyoshi E Katakura M Salivary immunoglobulin a secretion and polymeric ig receptor expression in the submandibular glands are enhanced in heat-acclimated rats. Int J Mol Sci. (2020) 21(3):815. 10.3390/ijms21030815 32012687 PMC7037029

[40] Arnold RR Ribeiro AA. Introduction to the oral cavity. In: Andrea Azcarate-Peril M Arnold RR Burno-Bárcena JM, editors. How Fermented Foods Feed a Healthy Gut Microbiota: A Nutrition Continuum. Cham, Switzerland: Springer (2019). p. 141–53.

[41] Frangeskou M Lopez-Valcarcel B Serra-Majem L. Dehydration in the elderly: a review focused on economic burden. J Nutr Health Aging. (2015) 19:619–27. 10.1007/s12603-015-0491-2 26054498 PMC12877650

[42] Walsh NP Montague JC Callow N Rowlands AV. Saliva flow rate, total protein concentration and osmolality as potential markers of whole body hydration status during progressive acute dehydration in humans. Arch Oral Biol. (2004) 49(2):149–54. 10.1016/j.archoralbio.2003.08.001 14693209

[43] Fortes MB Diment BC Di Felice U Walsh NP. Dehydration decreases saliva antimicrobial proteins important for mucosal immunity. Appl Physiol Nutr Metab. (2012 Oct) 37(5):850–9. 10.1139/h2012-054 22686429

[44] Dennesen P Van Der Ven A Vlasveld M Lokker L Ramsay G Kessels A Inadequate salivary flow and poor oral mucosal status in intubated intensive care unit patients. Crit Care Med. (2003) 31(3):781–6. 10.1097/01.CCM.0000053646.04085.29 12626984

[45] Affoo RH Foley N Garrick R Siqueira WL Martin RE. Meta-analysis of salivary flow rates in young and older adults. J Am Geriatr Soc. (2015) 63(10):2142–51. 10.1111/jgs.13652 26456531

[46] Gupta N Pal M Rawat S Grewal MS Garg H Chauhan D Radiation-induced dental caries, prevention and treatment-A systematic review. Natl J Maxillofac Surg. (2015) 6(2):160. 10.4103/0975-5950.183870 27390489 PMC4922225

[47] Sreebny LM Schwartz SS. A reference guide to drugs and dry mouth–2nd edition. Gerodontology. (1997) 14(1):33–47. 10.1111/j.1741-2358.1997.00033.x 9610301

[48] Percival RS Challacombe SJ Marsh PD. Flow rates of resting whole and stimulated parotid saliva in relation to age and gender. J Dent Res. (1994) 73(8):1416–20. 10.1177/00220345940730080401 8083437

[49] Dawes C Wong DT. Role of saliva and salivary diagnostics in the advancement of oral health. J Dent Res. (2019) 98(2):133–41. 10.1177/0022034518816961 30782091 PMC6900436

[50] Gupta A Epstein JB Sroussi H. Hyposalivation in elderly patients. J Can Dent Assoc. (2006) 72(9):841–6. 17109806

[51] Iwabuchi H Fujibayashi T Yamane GY Imai H Nakao H. Relationship between hyposalivation and acute respiratory infection in dental outpatients. Gerontology. (2012) 58(3):205–11. 10.1159/000333147 22104982

[52] Siqueira WL Salih E Wan DL Helmerhorst EJ Oppenheim FG. Proteome of human minor salivary gland secretion. J Dent Res. (2008) 87(5):445–50. 10.1177/154405910808700508 18434574 PMC2857507

[53] Leibovitz A Plotnikov G Habot B Rosenberg M Wolf A Nagler R Saliva secretion and oral flora in prolonged nasogastric tube-fed elderly patients. Isr Med Assoc J. (2003) 5(5):329–32. 12811948

[54] Kim IS Han TR. Influence of mastication and salivation on swallowing in stroke patients. Arch Phys Med Rehabil. (2005) 86(10):1986–90. 10.1016/j.apmr.2005.05.004 16213243

[55] Hoover E Aslam S Krishnamurthy K. Physiology, Sebaceous Glands. InStatPearls: StatPearls Publishing (2022). 29762994

[56] Bibi T Khurshid Z Rehman A Imran E Srivastava KC Shrivastava D. Gingival crevicular fluid (GCF): a diagnostic tool for the detection of periodontal health and diseases. Molecules. (2021) 26(5):1208. 10.3390/molecules26051208 33668185 PMC7956529

[57] Barros SP Williams R Offenbacher S Morelli T. Gingival crevicular as a source of biomarkers for periodontitis. Periodontol 2000. (2016) 70(1):53. 10.1111/prd.12107 26662482 PMC4911175

[58] Subbarao KC Nattuthurai GS Sundararajan SK Sujith I Joseph J Syedshah YP. Gingival crevicular fluid: an overview. J Pharm Bioallied Sci. (2019) 11(Suppl 2):S135. 10.4103/JPBS.JPBS_56_19 31198325 PMC6555362

[59] Farci F Soni A. Histology, tooth. In: StatPearls. Treasure Island (FL): StatPearls Publishing (2023) (updated July 4, 2022). p. 1–4. 34283421

[60] Talal A Hamid SK Khan M Khan AS. Structure of biological apatite: bone and tooth. In: Khan AS Chaudhry AA, editors. Handbook of Ionic Substituted Hydroxyapatites. Sawston, Cambridge: Woodhead Publishing (2020). p. 1–19.

[61] Loesche WJ. Microbiology of Dental Decay and Periodontal Disease. Medical Microbiology. 4th edn. Galveston, TX: The University of Texas Medical Branch at Galveston (1996). 21413316

[62] Duverger O Beniash E Morasso MI. Keratins as components of the enamel organic matrix. Matrix Biol. (2016) 52–54:260–5. 10.1016/j.matbio.2015.12.007 PMC4875797 26709044

[63] Abou Neel EA Aljabo A Strange A Ibrahim S Coathup M Young AM Demineralization–remineralization dynamics in teeth and bone. Int J Nanomed. (2016) 11:4743–63. 10.2147/IJN.S107624 PMC5034904 27695330

[64] Yamamoto T Hasegawa T Yamamoto T Hongo H Amizuka N. Histology of human cementum: its structure, function, and development. Jpn Dent Sci Rev. (2016) 52(3):63–74. 10.1016/j.jdsr.2016.04.002 28408958 PMC5390338

[65] Koussoulakou DS Margaritis LH Koussoulakos SL. A curriculum vitae of teeth: evolution, generation, regeneration. Int J Diol Sci. (2009) 5(3):226. 10.7150/ijbs.5.226 PMC2651620 19266065

[66] Torabi S Soni A. Histology, periodontium. In: StatPearls. Treasure Island, FL: StatPearls Publishing (2022). p. 1–5. 34033366

[67] Chen T Yu WH Izard J Baranova OV Lakshmanan A Dewhirst FE. The human oral microbiome database: a web accessible resource for investigating oral microbe taxonomic and genomic information. Database. (2010) 2010:1–10. 10.1093/database/baq013 PMC2911848 20624719

[68] Baty JJ Stoner SN Scoffield JA. Oral commensal streptococci: gatekeepers of the oral cavity. J Bacteriol. (2022) 204(11):e00257–22. 10.1128/jb.00257-22 36286512 PMC9664950

[69] Cugini C Ramasubbu N Tsiagbe VK Fine DH. Dysbiosis from a microbial and host perspective relative to oral health and disease. Front Microbiol. (2021) 12:617485. 10.3389/fmicb.2021.617485 33763040 PMC7982844

[70] Khan R Petersen FC Shekhar S. Commensal bacteria: an emerging player in defense against respiratory pathogens. Front Immunol. (2019) 10:1203. 10.3389/fimmu.2019.01203 31214175 PMC6554327

[71] Mark Welch JL Dewhirst FE Borisy GG. Biogeography of the oral microbiome: the site-specialist hypothesis. Annu Rev Microbiol. (2019) 73:335–58. 10.1146/annurev-micro-090817-062503 31180804 PMC7153577

[72] Segata N Haake SK Mannon P Lemon KP Waldron L Gevers D Composition of the adult digestive tract bacterial microbiome based on seven mouth surfaces, tonsils, throat and stool samples. Genome Biol. (2012) 13:1–8. 10.1186/gb-2012-13-6-r42 PMC3446314 22698087

[73] Sultan AS Kong EF Rizk AM Jabra-Rizk MA. The oral microbiome: a lesson in coexistence. PLoS Pathog. (2018) 14(1):e1006719. 10.1371/journal.ppat.1006719 29370304 PMC5784999

[74] Li X Kolltveit KM Tronstad L Olsen I. Systemic diseases caused by oral infection. Clin Microbiol Rev. (2000) 13(4):547–58. 10.1128/CMR.13.4.547 11023956 PMC88948

[75] Kaplan JÁ. Biofilm dispersal: mechanisms, clinical implications, and potential therapeutic uses. J Dent Res. (2010) 89(3):205–18. 10.1177/0022034509359403 20139339 PMC3318030

[76] Stoodley P Wilson S Hall-Stoodley L Boyle JD Lappin-Scott HM Costerton JW. Growth and detachment of cell clusters from mature mixed-species biofilms. Appl Environ Microbiol. (2001) 67(12):5608–13. 10.1128/AEM.67.12.5608-5613.2001 11722913 PMC93350

[77] Abdulkareem AA Al-Taweel FB Al-Sharqi AJ Gul SS Sha A Chapple IL. Current concepts in the pathogenesis of periodontitis: from symbiosis to dysbiosis. J Oral Microbiol. (2023) 15(1):2197779. 10.1080/20002297.2023.2197779 37025387 PMC10071981

[78] Oyetola EO Awosusi OO Agho ET Abdullahi MA Suleiman IK Egunjobi S. Salivary bacterial count and its implications on the prevalence of oral conditions. J Contemp Dent Pract. (2019 ) 20(2):184–9. 10.5005/jp-journals-10024-2495 31058633

[79] Paju S Scannapieco FA. Oral biofilms, periodontitis, and pulmonary infections. Oral Dis. (2007) 13(6):508–12. 10.1111/j.1601-0825.2007.01410a.x 17944664 PMC2258093

[80] Yu L Maishi N Akahori E Hasebe A Takeda R Matsuda AY The oral bacterium Streptococcus mutans promotes tumor metastasis by inducing vascular inflammation. Cancer Sci. (2022) 113(11):3980. 10.1111/cas.15538 35997541 PMC9633306

[81] Berger G Bitterman R Azzam ZS. The human microbiota: the rise of an “empire”. Rambam Maimonides Med J. (2015) 6(2):1–5. 10.5041/RMMJ.10202 PMC4422457 25973270

[82] Chow J Tang H Mazmanian SK. Pathobionts of the gastrointestinal microbiota and inflammatory disease. Curr Opin Immunol. (2011) 23(4):473–80. 10.1016/j.coi.2011.07.010 21856139 PMC3426444

[83] Dickson RP Erb-Downward JR Martinez FJ Huffnagle GB. The microbiome and the respiratory tract. Annu Rev Physiol. (2016) 78:481–504. 10.1146/annurev-physiol-021115-105238 26527186 PMC4751994

[84] Hilty M Burke C Pedro H Cardenas P Bush A Bossley C Disordered microbial communities in asthmatic airways. PloS One. (2010) 5(1):e8578. 10.1371/journal.pone.0008578 20052417 PMC2798952

[85] Dickson RP Erb-Downward JR Huffnagle GB. Towards an ecology of the lung: new conceptual models of pulmonary microbiology and pneumonia pathogenesis. Lancet Respir Med. (2014) 2(3):238–46. 10.1016/S2213-2600(14)70028-1 24621685 PMC4004084

[86] Dickson RP Erb-Downward JR Freeman CM McCloskey L Falkowski NR Huffnagle GB Bacterial topography of the healthy human lower respiratory tract. mBio. (2017) 8(1):10–128. 10.1128/mBio.02287-16 PMC5312084 28196961

[87] Min Z Yang L Hu Y Huang R. Oral microbiota dysbiosis accelerates the development and onset of mucositis and oral ulcers. Front Microbiol. (2023) 14:1061032. 10.3389/fmicb.2023.1061032 36846768 PMC9948764

[88] Coulthwaite L Verran J. Potential pathogenic aspects of denture plaque. Br J Biomed Sci. (2007) 64(4):180–9. 10.1080/09674845.2007.11732784 18236742

[89] El-Solh AA. Association between pneumonia and oral care in nursing home residents. Lung. (2011) 189:173–80. 10.1007/s00408-011-9297-0 21533635

[90] Ewan VC Sails AD Walls AW Rushton S Newton JL. Dental and microbiological risk factors for hospital-acquired pneumonia in non-ventilated older patients. PLoS One. (2015) 10(4):e0123622. 10.1371/journal.pone.0123622 25923662 PMC4414413

[91] Drinka P. Preventing aspiration in the nursing home: the role of biofilm and data from the ICU. J Am Med Dir Assoc. (2010) 11(1):70–7. 10.1016/j.jamda.2009.03.020 20129217

[92] Donlan RM Costerton JW. Biofilms: survival mechanisms of clinically relevant microorganisms. Clin Microbiol Rev. (2002) 15(2):167–93. 10.1128/CMR.15.2.167-193.2002 11932229 PMC118068

[93] Sauer K Camper AK Ehrlich GD Costerton JW Davies DG. Pseudomonas aeruginosa displays multiple phenotypes during development as a biofilm. J Bacteriol. (2002) 184(4):1140–54. 10.1128/jb.184.4.1140-1154.2002 11807075 PMC134825

[94] Kilian M Chapple IL Hannig M Marsh PD Meuric V Pedersen AM The oral microbiome—an update for oral healthcare professionals. Br Dent J. (2016) 221(10):657–66. 10.1038/sj.bdj.2016.865 27857087

[95] Rudney JD. Saliva and dental plaque. Adv Dent Res. (2000) 14(1):29–39. 10.1177/08959374000140010401 11842921

[96] Rowshani B Timmerman MF Van der Velden U. Plaque development in relation to the periodontal condition and bacterial load of the saliva. J Clin Periodontol. (2004) 31(3):214–8. 10.1111/j.0303-6979.2004.00468.x 15016026

[97] Sabharwal A Stellrecht E Scannapieco FA. Associations between dental caries and systemic diseases: a scoping review. BMC Oral Health. (2021) 21:1–35. 10.1186/s12903-021-01803-w 34563194 PMC8466895

[98] Selwitz RH Ismail AI Pitts NB. Dental caries. Lancet. (2007) 369(9555):51–9. 10.1016/S0140-6736(07)60031-2 17208642

[99] Lemos JA Palmer SR Zeng L Wen ZT Kajfasz JK Freires IA The biology of Streptococcus mutans. Microbiol Spectr. (2019) 7(1):10–128. 10.1128/microbiolspec.GPP3-0051-2018 PMC6615571 30657107

[100] Centers for Disease Control and Prevention. Hygiene-related Diseases. Atlanta (GA): CDC (2014).

[101] Heng CC. Tooth decay is the most prevalent disease. Fed Pract. (2016) 33(10):31. 30766141 PMC6373711

[102] Featherstone JD. The science and practice of caries prevention. J Am Dent Assoc. (2000) 131(7):887–99. 10.14219/jada.archive.2000.0307 10916327

[103] Loesche WJ. Role of Streptococcus mutans in human dental decay. Microbiol Rev. (1986) 50(4):353–80. 10.1128/mr.50.4.353-380.1986 3540569 PMC373078

[104] Dye BA Thornton-Evans G Li X Iafolla T. Dental Caries and Tooth Loss in Adults in the United States, 2011–2012. Hyattsville, MA, USA: US Department of Health and Human Services, Centers for Disease Control and Prevention, National Center for Health Statistics (2015).

[105] Terpenning MS Taylor GW Lopatin DE Kerr CK Dominguez BL Loesche WJ. Aspiration pneumonia: dental and oral risk factors in an older veteran population. J Am Geriatr Soc. (2001) 49(5):557–63. 10.1046/j.1532-5415.2001.49113.x 11380747

[106] Scannapieco FA Mylotte JM. Relationships between periodontal disease and bacterial pneumonia. J Periodontol. (1996) 67:1114–22. 10.1902/jop.1996.67.10s.1114 8910830

[107] Coventry J Griffiths G Scully C Tonetti M. Periodontal disease. Br Med J. (2000) 321(7252):36–9. 10.1136/bmj.321.7252.36 10875835 PMC1127686

[108] Kinane DF. Causation and pathogenesis of periodontal disease. Periodontol 2000. (2001) 25(1):8–20. 10.1034/j.1600-0757.2001.22250102.x 11155179

[109] Usui M Onizuka S Sato T Kokabu S Ariyoshi W Nakashima K. Mechanism of alveolar bone destruction in periodontitis—periodontal bacteria and inflammation. Jpn Dent Sci Rev. (2021) 57:201–8. 10.1016/j.jdsr.2021.09.005 34703508 PMC8524191

[110] Eke PI Thornton-Evans GO Wei L Borgnakke WS Dye BA Genco RJ. Periodontitis in US adults: national health and nutrition examination survey 2009–2014. J Am Dent Assoc. (2018) 149(7):576–88. 10.1016/j.adaj.2018.04.023 29957185 PMC8094373

[111] Sanz M D'Aiuto F Deanfield J Fernandez-Avilés F. European Workshop in periodontal health and cardiovascular disease—scientific evidence on the association between periodontal and cardiovascular diseases: a review of the literature. Eur Heart J Suppl. (2010) 12(suppl_B):B3–12. 10.1093/eurheartj/suq003

[112] Bartold PM Van Dyke TE. Periodontitis: a host-mediated disruption of microbial homeostasis. Unlearning learned concepts. Periodontol 2000. (2013) 62(1):203–17. 10.1111/j.1600-0757.2012.00450.x 23574467 PMC3692012

[113] Radaic A Kapila YL. The oralome and its dysbiosis: new insights into oral microbiome-host interactions. Comput Struct Biotechnol J. (2021) 19:1335–60. 10.1016/j.csbj.2021.02.010 33777334 PMC7960681

[114] Hajishengallis G. The inflammophilic character of the periodontitis-associated microbiota. Mol Oral Microbiol. (2014) 29(6):248–57. 10.1111/omi.12065 24976068 PMC4232466

[115] Van Dyke TE Bartold PM Reynolds EC. The nexus between periodontal inflammation and dysbiosis. Front Immunol. (2020) 11:511. 10.3389/fimmu.2020.00511 32296429 PMC7136396

[116] Cekici A Kantarci A Hasturk H Van Dyke TE. Inflammatory and immune pathways in the pathogenesis of periodontal disease. Periodontol 2000. (2014) 64(1):57–80. 10.1111/prd.12002 24320956 PMC4500791

[117] Trombelli L Farina R Silva CO Tatakis DN. Plaque-induced gingivitis: case definition and diagnostic considerations. J Periodontol. (2018) 45:S44–67. 10.1111/jcpe.12939 29926492

[118] Brecx MC Fröhlicher I Gehr P Lang NP. Stereological observations on long-term experimental gingivitis in man. J Clin Periodontol. (1988) 15(10):621–7. 10.1111/j.1600-051X.1988.tb02262.x 3198779

[119] Page RC Schroeder HE. Pathogenesis of inflammatory periodontal disease. A summary of current work. Lab Invest. (1976) 34(3):235–49. 765622

[120] Hajishengallis G Korostoff JM. Revisiting the page & schroeder model: the good, the bad and the unknowns in the periodontal host response 40 years later. Periodontol 2000. (2017) 75(1):116–51. 10.1111/prd.12181 28758305 PMC5539911

[121] Peng X Cheng L You Y Tang C Ren B Li Y Oral microbiota in human systematic diseases. Int J Oral Sci. (2022) 14(1):14. 10.1038/s41368-022-00163-7 35236828 PMC8891310

[122] Hajishengallis G Chavakis T. Local and systemic mechanisms linking periodontal disease and inflammatory comorbidities. Nat Rev Immunol. (2021) 21(7):426–40. 10.1038/s41577-020-00488-6 33510490 PMC7841384

[123] Garcia RI Henshaw MM Krall EA. Relationship between periodontal disease and systemic health. Periodontol 2000. (2001) 25(1):21–36. 10.1034/j.1600-0757.2001.22250103.x 11155180

[124] Kim J Amar S. Periodontal disease and systemic conditions: a bidirectional relationship. Odontology. (2006) 94:10–21. 10.1007/s10266-006-0060-6 16998613 PMC2443711

[125] Hegde R Awan KH. Effects of periodontal disease on systemic health. Dis Mon. (2019) 65(6):185–92. 10.1016/j.disamonth.2018.09.011 30384973

[126] Bansal M Khatri M Taneja V. Potential role of periodontal infection in respiratory diseases-a review. J Med Life. (2013) 6(3):244. 24155782 PMC3786481

[127] Grossi SG Genco RJ. Periodontal disease and diabetes mellitus: a two-way relationship. Ann Periodontology. (1998) 3(1):51–61. 10.1902/annals.1998.3.1.51 9722690

[128] Libby P Ridker PM Maseri A. Inflammation and atherosclerosis. Circulation. (2002) 105(9):1135–43. 10.1161/hc0902.104353 11877368

[129] Scannapieco FA. Role of oral bacteria in respiratory infection. J Periodontol. (1999) 70(7):793–802. 10.1902/jop.1999.70.7.793 10440642

[130] Scannapieco FA Bush RB Paju S. Associations between periodontal disease and risk for nosocomial bacterial pneumonia and chronic obstructive pulmonary disease. A systematic review. Ann Periodontol. (2003) 8(1):54–69. 10.1902/annals.2003.8.1.54 14971248

[131] Awano S Ansai T Takata Y Soh I Akifusa S Hamasaki T Oral health and mortality risk from pneumonia in the elderly. J Dent Res. (2008) 87(4):334–9. 10.1177/154405910808700418 18362314

[132] Holmstrup P Damgaard C Olsen I Klinge B Flyvbjerg A Nielsen CH Comorbidity of periodontal disease: two sides of the same coin? An introduction for the clinician. J Oral Microbiol. (2017) 9(1):1332710. 10.1080/20002297.2017.1332710 28748036 PMC5508374

[133] Gomes-Filho IS Passos JS Seixas da Cruz S. Respiratory disease and the role of oral bacteria. J Oral Microbiol. (2010) 2(1):5811. 10.3402/jom.v2i0.5811 PMC3084574 21523216

[134] Kouanda B Sattar Z Geraghty P. Periodontal diseases: major exacerbators of pulmonary diseases? Pulm Med. (2021) 2021:1–10. 10.1155/2021/4712406 PMC8577952 34765263

[135] Wu Z Xiao C Chen F Wang Y Guo Z. Pulmonary disease and periodontal health: a meta-analysis. Sleep Breath. (2022) 26(4):1857–68. 10.1007/s11325-022-02577-3 35122603

[136] Claydon NC. Current concepts in toothbrushing and interdental cleaning. Periodontol 2000. (2008) 48(1):10–22. 10.1111/j.1600-0757.2008.00273.x 18715352

[137] American Dental Association. Brushing Your Teeth. Chicago: American Dental Association (2023). Available online at: https://www.mouthhealthy.org/all-topics-a-z/brushing-your-teeth (cited 2012).

[138] Attin T Hornecker E. Tooth brushing and oral health: how frequently and when should tooth brushing be performed? Oral Health Prev Dent. (2005) 3(3):135–40. 16355646

[139] Buglass EA. Oral hygiene. Br J Nurs. (1995) 4(9):516–9. 10.12968/bjon.1995.4.9.516 7599497

[140] Quagliarello V Ginter S Han L Van Ness P Allore H Tinetti M. Modifiable risk factors for nursing home-acquired pneumonia. Clin Infect Dis. (2005) 40(1):1–6. 10.1086/426023 15614684

[141] Sjögren P Nilsson E Forsell M Johansson O Hoogstraate J. A systematic review of the preventive effect of oral hygiene on pneumonia and respiratory tract infection in elderly people in hospitals and nursing homes: effect estimates and methodological quality of randomized controlled trials. J Am Geriatr Soc. (2008) 56(11):2124–30. 10.1111/j.1532-5415.2008.01926.x 18795989

[142] Todd JT Stuart A Lintzenich CR Wallin J Grace-Martin K Butler SG. Stability of aspiration status in healthy adults. Ann Otol Rhinol Laryngol. (2013) 122(5):289–93. 10.1177/000348941312200501 23815044

[143] Giuliano KK Penoyer D Middleton A Baker D. Oral care as prevention for nonventilator hospital-acquired pneumonia: a four-unit cluster randomized study. Am J Nurs. (2021) 121(6):24–33. 10.1097/01.NAJ.0000753468.99321.93 33993136

[144] Khadka S Khan S King A Goldberg LR Crocombe L Bettiol S. Poor oral hygiene, oral microorganisms and aspiration pneumonia risk in older people in residential aged care: a systematic review. Age Ageing. (2021) 50(1):81–7. 10.1093/ageing/afaa102 32677660

[145] Fields LB. Oral care intervention to reduce incidence of ventilator-associated pneumonia in the neurologic intensive care unit. J Neurosci Nurs. (2008) 40(5):291–8. 10.1097/01376517-200810000-00007 18856250

[146] Scannapieco FA Yu J Raghavendran K Vacanti A Owens SI Wood K A randomized trial of chlorhexidine gluconate on oral bacterial pathogens in mechanically ventilated patients. Crit Care. (2009) 13(4):1–2. 10.1186/cc7967 PMC2750165 19765321

[147] Munro CL Grap MJ Jones DJ McClish DK Sessler CN. Chlorhexidine, toothbrushing, and preventing ventilator-associated pneumonia in critically ill adults. Am J Crit Care. (2009) 18(5):428–37. 10.4037/ajcc2009792 19723863 PMC3722581

[148] Pobo A Lisboa T Rodriguez A Sole R Magret M Trefler S A randomized trial of dental brushing for preventing ventilator-associated pneumonia. Chest. (2009) 136(2):433–9. 10.1378/chest.09-0706 19482956

[149] Saddki N Mohamad Sani FE Tin-Oo MM. Oral care for intubated patients: a survey of intensive care unit nurses. Nurs Crit Care. (2017) 22(2):89–98. 10.1111/nicc.12119 25349099

[150] Alhazzani W Smith O Muscedere J Medd J Cook D. Toothbrushing for critically ill mechanically ventilated patients: a systematic review and meta-analysis of randomized trials evaluating ventilator-associated pneumonia. Crit Care Med. (2013) 41(2):646–55. 10.1097/CCM.0b013e3182742d45 23263588

[151] de Lacerda Vidal CF Vidal AK Monteiro JG Cavalcanti A Henriques AP Oliveira M Impact of oral hygiene involving toothbrushing versus chlorhexidine in the prevention of ventilator-associated pneumonia: a randomized study. BMC Infect Dis. (2017) 17:1–9. 10.1186/s12879-017-2188-0 28143414 PMC5286780

[152] Hopcraft MS Morgan MV Satur JG Wright FC Darby IB. Oral hygiene and periodontal disease in victorian nursing homes. Gerodontology. (2012) 29(2):e220–8. 10.1111/j.1741-2358.2010.00448.x 21083744

[153] De Visschere L de Baat C Schols JM Deschepper E Vanobbergen J. Evaluation of the implementation of an ‘oral hygiene protocol’in nursing homes: a 5-year longitudinal study. Community Dent Oral Epidemiol. (2011) 39(5):416–25. 10.1111/j.1600-0528.2011.00610.x 21362011

[154] D'Aiuto F Gable D Syed Z Allen Y Wanyonyi KL White S Evidence summary: the relationship between oral diseases and diabetes. Br Dent J. (2017) 222(12):944–8. 10.1038/sj.bdj.2017.544 28642531

[155] Islas-Granillo H Casanova-Rosado JF de la Rosa-Santillana R Casanova-Rosado AJ Islas-Zarazúa R de Lourdes Márquez-Corona M Self-reported oral hygiene practices with emphasis on frequency of tooth brushing: a cross-sectional study of Mexican older adults aged 60 years or above. Med. (2020) 99(36):e21622. 10.1097/MD.0000000000021622 PMC7478503 32898997

[156] Coleman P Watson NM. Oral care provided by certified nursing assistants in nursing homes. J Am Geriatr Soc. (2006) 54(1):138–43. 10.1111/j.1532-5415.2005.00565.x 16420211

[157] Wagner SR Eriksen CL Hede B Christensen LB. Toothbrushing compliance tracking in a nursing home setting using telemonitoring-enabled powered toothbrushes. Br Dent J. (2021):1–6. 10.1038/s41415-021-3169-7 34239058

[158] Gurgel-Juarez N Mallet K Egan M Blacquiere D Laneville A Perrier MF Oral care in acute stroke. Perspectives of the ASHA Special Interest Groups. (2022) 7(1):165–73. 10.1044/2021_PERSP-21-00108

[159] Yaacob M Worthington HV Deacon SA Deery C Walmsley AD Robinson PG Powered versus manual toothbrushing for oral health. Cochrane Database of Syst Rev. (2014) 6:1–116. 10.1002/14651858.CD002281.pub3 PMC7133541 24934383

[160] Elkerbout TA Slot DE Rosema NM Van der Weijden GA. How effective is a powered toothbrush as compared to a manual toothbrush? A systematic review and meta-analysis of single brushing exercises. Int J Dent Hyg. (2020) 18(1):17–26. 10.1111/idh.12401 31050195 PMC7004084

[161] Rosema NA Slot DE van Palenstein Helderman WH Wiggelinkhuizen L Van der Weijden GA. The efficacy of powered toothbrushes following a brushing exercise: a systematic review. Int J Dent Hyg. (2016) 14(1):29–41. 10.1111/idh.12115 25545231

[162] Re D Augusti G Battaglia D Giannì AB Augusti D. Is a new sonic toothbrush more effective in plaque removal than a manual toothbrush. Eur J Paediatr Dent. (2015) 16(1):13–8. 10.1007/s40368-014-0139-7 25793947

[163] Lavigne SE Doupe MB Iacopino AM Mahmud S Elliott L. The effects of power toothbrushing on periodontal inflammation in a Canadian nursing home population: a randomized controlled trial. Int J Dent Hyg. (2017) 15(4):328–34. 10.1111/idh.12268 28105737

[164] Wang P Xu Y Zhang J Chen X Liang W Liu X Comparison of the effectiveness between power toothbrushes and manual toothbrushes for oral health: a systematic review and meta-analysis. Acta Odontol Scand. (2020) 78(4):265–74. 10.1080/00016357.2019.1697826 32285744

[165] Fjeld KG Eide H Mowe M Sandvik L Willumsen T. A 1-year follow-up of a randomized clinical trial with focus on manual and electric toothbrushes’ effect on dental hygiene in nursing homes. Acta Odontol Scand. (2018) 76(4):257–61. 10.1080/00016357.2017.1416166 29239260

[166] Niederman R. Manual versus powered toothbrushes: the cochrane review. J Am Dent Assoc. (2003) 134(9):1240–4. 10.14219/jada.archive.2003.0359 14528996

[167] Verma S Bhat KM. Acceptability of powered toothbrushes for elderly individuals. J Public Health Dent. (2004) 64(2):115–7. 10.1111/j.1752-7325.2004.tb02738.x 15180082

[168] Grap MJ Munro CL Ashtiani B Bryant S. Oral care interventions in critical care: frequency and documentation. Am J Crit Care. (2003) 12(2):113–8. 10.4037/ajcc2003.12.2.113 12625169

[169] Huang ST Chiou CC Liu HY. Risk factors of aspiration pneumonia related to improper oral hygiene behavior in community dysphagia persons with nasogastric tube feeding. J Dent Sci. (2017) 12(4):375–81. 10.1016/j.jds.2017.06.001 30895078 PMC6395351

[170] Wanyonyi C Suila J. Best practice in basic oral care among cancer patients. (2015). p. 1–2(7). Available online at: https://www.theseus.fi/bitstream/handle/10024/101593/Wanyonyi_Celestine.pdf?sequence=1 (Accessed March 27, 2024).

[171] Ikeda M Miki T Atsumi M Inagaki A Mizuguchi E Meguro M Effective elimination of contaminants after oral care in elderly institutionalized individuals. Geriatr Nurs. (2014) 35(4):295–9. 10.1016/j.gerinurse.2014.03.003 24755196

[172] Wadsworth A. Mouth disinfection in the prophylaxis and treatment of pneumonia. J Infect Dis. (1906) 3:774–97. 10.1093/infdis/3.5.774

[173] Radzki D Wilhelm-Węglarz M Pruska K Kusiak A Ordyniec-Kwaśnica I. A fresh Look at mouthwashes—what is inside and what is it for? Int J Environ Res Public Health. (2022) 19(7):3926. 10.3390/ijerph19073926 35409608 PMC8997378

[174] Patil SS Yadav AR Chopade A Mohite S. Design, development and evaluation of herbal mouthwash for antibacterial potency against oral bacteria. J Univ Shanghai SciTechnol. (2020) 22(11):881–98.

[175] Susanto H. Xerostomia severity difference between elderly using alcohol and non alcohol-containing mouthwash. Dent J (Majalah Kedokteran Gigi). (2015) 48(3):109–12. 10.20473/j.djmkg.v48.i3.p109-112

[176] Ustrell-Borràs M Traboulsi-Garet B Gay-Escoda C. Alcohol-based mouthwash as a risk factor of oral cancer: a systematic review. Medicina oral. Patologia Oral y Cirugia Bucal. (2020) 25(1):e1–12. 10.4317/medoral.23085 PMC6982979 31655832

[177] Varoni E Tarce M Lodi G Carrassi A. Chlorhexidine (CHX) in dentistry: state of the art. Minerva Stomatol. (2012) 61(9):399–419. 22976567

[178] Chastre J Fagon JY. Ventilator-associated pneumonia. Am J Respir Crit Care Med. (2002) 165(7):867–903. 10.1164/ajrccm.165.7.2105078 11934711

[179] Berry AM Davidson PM Masters J Rolls K Ollerton R. Effects of three approaches to standardized oral hygiene to reduce bacterial colonization and ventilator associated pneumonia in mechanically ventilated patients: a randomised control trial. Int J Nurs Stud. (2011) 48(6):681–8. 10.1016/j.ijnurstu.2010.11.004 21185559

[180] Chan EY Ruest A Meade MO Cook DJ. Oral decontamination for prevention of pneumonia in mechanically ventilated adults: systematic review and meta-analysis. Br Med J. (2007) 334(7599):889. 10.1136/bmj.39136.528160.BE 17387118 PMC1857782

[181] Bergmans DC Bonten MJ Gaillard CA Paling JC van der Geest SI van Tiel FH Prevention of ventilator-associated pneumonia by oral decontamination: a prospective, randomized, double-blind, placebo-controlled study. Am J Respir Crit Care Med. (2001) 164(3):382–8. 10.1164/ajrccm.164.3.2005003 11500337

[182] Kollef M Pittet D Sánchez García M Chastre J Fagon JY Bonten M A randomized double-blind trial of iseganan in prevention of ventilator-associated pneumonia. Am J Respir Crit Care Med. (2006) 173(1):91–7. 10.1164/rccm.200504-656OC 16192451

[183] Laggner AN Tryba M Georgopoulos A Lenz K Grimm G Graninger W Oropharyngeal decontamination with gentamycin for long-term ventilated patients on stress ulcer prophylaxis with sucralfate? Wien Klin Wochenschr. (1994) 106:15–9. 8135026

[184] Fourrier F Dubois D Pronnier P Herbecq P Leroy O Desmettre T Effect of gingival and dental plaque antiseptic decontamination on nosocomial infections acquired in the intensive care unit: a double-blind placebo-controlled multicenter study. Crit Care Med. (2005) 33(8):1728–35. 10.1097/01.CCM.0000171537.03493.B0 16096449

[185] Seguin P Tanguy M Laviolle B Tirel O Mallédant Y. Effect of oropharyngeal decontamination by povidone-iodine on ventilator-associated pneumonia in patients with head trauma. Crit Care Med. (2006) 34(5):1514–9. 10.1097/01.CCM.0000214516.73076.82 16540962

[186] DeRiso AJ II Ladowski JS Dillon TA Justice JW Peterson AC. Chlorhexidine gluconate 0.12% oral rinse reduces the incidence of total nosocomial respiratory infection and nonprophylactic systemic antibiotic use in patients undergoing heart surgery. Chest. (1996) 109(6):1556–61. 10.1378/chest.109.6.1556 8769511

[187] Koeman M Van Der Ven AJ Hak E Joore HC Kaasjager K de Smet AG Oral decontamination with chlorhexidine reduces the incidence of ventilator-associated pneumonia. Am J Respir Crit Care Med. (2006) 173(12):1348–55. 10.1164/rccm.200505-820OC 16603609

[188] Segers P Speekenbrink RG Ubbink DT van Ogtrop ML de Mol BA. Prevention of nosocomial infection in cardiac surgery by decontamination of the nasopharynx and oropharynx with chlorhexidine gluconate: a randomized controlled trial. JAMA. (2006) 296(20):2460–6. 10.1001/jama.296.20.2460 17119142

[189] MacNaughton PD Bailey J Donlin N Branfield P Williams A Rowswell H. A randomised controlled trial assessing the efficacy of oral chlorhexidine in ventilated patients. Intensive Care Med. (2004) 30(Suppl 1):S12.

[190] Beraldo CC Andrade DD. Oral hygiene with chlorhexidine in preventing pneumonia associated with mechanical ventilation. J Brasi Pneumol. (2008) 34:707–14. 10.1590/S1806-37132008000900012 18982209

[191] Goutham BS Manchanda K De Sarkar A Prakash R Jha K Mohammed S. Efficacy of two commercially available oral rinses-chlorhexidine and listerine on plaque and gingivitis-A comparative study. J Int Oral Health. (2013) 5(4):56. 24155621 PMC3780369

[192] Villar CC Pannuti CM Nery DM Morillo CM Carmona MJ Romito GA. Effectiveness of intraoral chlorhexidine protocols in the prevention of ventilator-associated pneumonia: meta-analysis and systematic review. Respir Care. (2016) 61(9):1245–59. 10.4187/respcare.04610 27507174

[193] Kocaçal Güler E Türk G. Oral chlorhexidine against ventilator-associated pneumonia and microbial colonization in intensive care patients. West J Nurs Res. (2019) 41(6):901–19. 10.1177/0193945918781531 29907077

[194] Keykha A Ramezani M Amini S Moonaghi HK. Oropharyngeal decontamination for prevention of VAP in patients admitted to intensive care units: a systematic review. J Caring Sci. (2022) 11(3):178. 36247039 10.34172/jcs.2021.029 PMC9526792

[195] Pineda LA Saliba RG El Solh AA. Effect of oral decontamination with chlorhexidine on the incidence of nosocomial pneumonia: a meta-analysis. Crit Care. (2006) 10(1):1–6. 10.1186/cc4837 PMC1550809 16507165

[196] Price R MacLennan G Glen J. Selective digestive or oropharyngeal decontamination and topical oropharyngeal chlorhexidine for prevention of death in general intensive care: systematic review and network meta-analysis. Br Med J. (2014) 348:1–15. 10.1136/bmj.g2197 PMC3970764 24687313

[197] Plantinga NL Wittekamp BH Leleu K Depuydt P Van den Abeele AM Brun-Buisson C Oral mucosal adverse events with chlorhexidine 2% mouthwash in ICU. Intensive Care Med. (2016) 42:620–1. 10.1007/s00134-016-4217-7 26850333 PMC5413521

[198] Bouadma L Klompas M. Oral care with chlorhexidine: beware!. Intensive Care Med. (2018) 44:1153–5. 10.1007/s00134-018-5221-x 29808343

[199] Alshehri FA. The use of mouthwash containing essential oils (LISTERINE®) to improve oral health: a systematic review. Saudi Dent J. (2018) 30(1):2–6. 10.1016/j.sdentj.2017.12.004 30166864 PMC6112363

[200] Vlachojannis C Winsauer H Chrubasik S. Effectiveness and safety of a mouthwash containing essential oil ingredients. Phytother Res. (2013) 27(5):685–91. 10.1002/ptr.4762 22761009

[201] Charles CH Mostler KM Bartels LL Mankodi SM. Comparative antiplaque and antigingivitis effectiveness of a chlorhexidine and an essential oil mouthrinse: 6-month clinical trial. J Clin Periodontol. (2004) 31(10):878–84. 10.1111/j.1600-051X.2004.00578.x 15367192

[202] https://www.cms.gov/medicare/quality-initiatives-patient-assessment-instruments/nursinghomequalityinits/downloads/mds20rai1202ch4.pdf.

[203] Chalmers J Johnson V. Evidence-based protocol: oral hygiene care for functionally dependent and cognitively impaired older adults. J Gerontol Nurs. (2004) 30(11):5–9. 10.3928/0098-9134-20041101-06 15575186

[204] Slade GD Spencer AJ. Development and evaluation of the oral health impact profile. Community Dent Health. (1994) 11(1):3–11. 8193981

[205] Santos CM Oliveira BH Nadanovsky P Hilgert JB Celeste RK Hugo FN. The oral health impact profile-14: a unidimensional scale? Cadernos de Saúde Pública. (2013) 29:749–757. 10.1590/s0102-311x2013000800012 23568304

[206] Campos LA Peltomäki T Marôco J Campos JA. Use of oral health impact profile-14 (OHIP-14) in different contexts. What is being measured? Int J Environ Res Public Health. (2021) 18(24):13412. 10.3390/ijerph182413412 34949018 PMC8703465

[207] Kayser-Jones J Bird WF Paul SM Long L Schell ES. An instrument to assess the oral health status of nursing home residents. Gerontologist. (1995) 35(6):814–24. 10.1093/geront/35.6.814 8557208

[208] Chalmers JM King PL Spencer AJ Wright FA Carter KD. The oral health assessment tool—validity and reliability. Aust Dent J. (2005) 50(3):191–9. 10.1111/j.1834-7819.2005.tb00360.x 16238218

[209] Maeda K Mori N. Poor oral health and mortality in geriatric patients admitted to an acute hospital: an observational study. BMC Geriatr. (2020) 20:1–7. 10.1186/s12877-020-1429-z PMC6986081 31992227

[210] Simpelaere IS Van Nuffelen G Vanderwegen J Wouters K De Bodt M. Oral health screening: feasibility and reliability of the oral health assessment tool as used by speech pathologists. Int Dent J. (2016) 66(3):178–89. 10.1111/idj.12220 26853437 PMC9376655

[211] Salamone K Yacoub E Mahoney AM Edward KL. Oral care of hospitalised older patients in the acute medical setting. Nurs Res Pract. (2013) 2013:1–4. 10.1155/2013/827670 PMC3683489 23819046

[212] White R. Nurse assessment of oral health: a review of practice and education. Br J Nurs. (2000) 9(5):260–6. 10.12968/bjon.2000.9.5.6359 11042780

[213] Yoneyama T Yoshida M Matsui T Sasaki H. Oral care and pneumonia. Lancet. (1999) 354(9177):515. 10.1016/S0140-6736(05)75550-1 10465203

[214] Ishikawa S Yamamori I Takamori S Kitabatake K Edamatsu K Sugano A Evaluation of effects of perioperative oral care intervention on hospitalization stay and postoperative infection in patients undergoing lung cancer intervention. Support Care Cancer. (2021) 29:135–43. 10.1007/s00520-020-05450-9 32323001

[215] Müller F. Oral hygiene reduces the mortality from aspiration pneumonia in frail elders. J Dent Res. (2015) 94(3_suppl):14S–6S. 10.1177/0022034514552494 25294365 PMC4541086

[216] Fiske J Griffiths J Jamieson R Manger D. Guidelines for oral health care for long-stay patients and residents. Gerodontology. (2000) 17(1):55–64. 10.1111/j.1741-2358.2000.00055.x 11203515

[217] Yoon MN Steele CM. Health care professionals’ perspectives on oral care for long-term care residents: nursing staff, speech–language pathologists and dental hygienists. Gerodontology. (2012) 29(2):e525–35. 10.1111/j.1741-2358.2011.00513.x 22462684

[218] Daniel BT Damato KL Johnson J. Educational issues in oral care. In: Burbage D, editor. Seminars in Oncology Nursing. Amsterdam: WB Saunders (2004). Vol. 20, no. 1, p. 48–52. 10.1053/j.soncn.2003.10.008 15038517

[219] Veerasamy A Lyons K Crabtree I Brunton P. Knowledge of nursing graduates on oral health care for older people in the long-term care. J Dent Educ. (2022) 86(7):830–8. 10.1002/jdd.12895 35129837

[220] Dahm TS Bruhn A LeMaster M. Oral care in the long-term care of older patients: how can the dental hygienist meet the need? American Dental Hygienists’ Association. (2015) 89(4):229–37. 26304947

[221] El-Solh AA Vora H Knight PR III Porhomayon J. Diagnostic utility of serum procalcitonin levels in pulmonary aspiration syndromes. Crit Care Med. (2011) 39(6):1251. 10.1097/CCM.0b013e31820a942c 21283001 PMC3102149

[222] Doshi M Mann J Quentin L Morton-Holtham L Eaton KA. Mouth care training and practice: a survey of nursing staff working in national health service hospitals in England. J Res Nurs. (2021) 26(6):574–90. 10.1177/17449871211016524 35265164 PMC8899309

[223] Wårdh I Jonsson M Wikström M. Attitudes to and knowledge about oral health care among nursing home personnel–an area in need of improvement. Gerodontology. (2012) 29(2):e787–92. 10.1111/j.1741-2358.2011.00562.x 21950522

[224] Palmers E Janssens L Phlypo I Vanhaecht K De Almeida Mello J De Visschere L Perceptions on oral care needs, barriers, and practices among managers and staff in long-term care settings for older people in Flanders, Belgium: a cross-sectional survey. Innovation in Aging. (2022) 6(5):igac046. 10.1093/geroni/igac046 36081406 PMC9447852

[225] Gammack JK Pulisetty S. Nursing education and improvement in oral care delivery in long-term care. J Am Med Dir Assoc. (2009) 10(9):658–61. 10.1016/j.jamda.2009.09.001 19883890

[226] Samson H Berven L Strand GV. Long-term effect of an oral healthcare programme on oral hygiene in a nursing home. Eur J Oral Sci. (2009) 117(5):575–9. 10.1111/j.1600-0722.2009.00673.x 19758255

[227] Ildarabadi EH Armat MR Motamedosanaye V Ghanei F. Effect of oral health care program on oral health status of elderly people living in nursing homes: a quasi-experimental study. Mater Sociomed. (2017) 29(4):263. 10.5455/msm.2017.29.263-267 29284996 PMC5723168

[228] Zimmerman S Sloane PD Cohen LW Barrick AL. Changing the culture of mouth care: mouth care without a battle. Gerontologist. (2014) 54(Suppl_1):S25–34. 10.1093/geront/gnt145 24443603

[229] Zimmerman S Sloane PD Ward K Wretman CJ Stearns SC Poole P Effectiveness of a mouth care program provided by nursing home staff vs standard care on reducing pneumonia incidence: a cluster randomized trial. JAMA Network Open. (2020) 3(6):e204321. 10.1001/jamanetworkopen.2020.4321 32558913 PMC7305523

[230] Jones H Newton JT Bower EJ. A survey of the oral care practices of intensive care nurses. Intensive Crit Care Nurs. (2004) 20(2):69–76. 10.1016/j.iccn.2004.01.004 15072774

[231] Sreenivasan VP Ganganna A Rajashekaraiah PB. Awareness among intensive care nurses regarding oral care in critically ill patients. J Indian Soc Periodontol. (2018) 22(6):541. 10.4103/jisp.jisp_30_18 30631234 PMC6305093

[232] Luk JK Chan DK. Preventing aspiration pneumonia in older people: do we have the ‘know-how’? Hong Kong Med J. (2014) 20(5):421. 10.12809/hkmj144251 24993858

[233] Koichiro UE. Preventing aspiration pneumonia by oral health care. Jpn Med Assoc J. (2011) 54(1):39–43.

[234] Blumenstein I Shastri YM Stein J. Gastroenteric tube feeding: techniques, problems and solutions. World J Gastroenterol. (2014) 20(26):8505. 10.3748/wjg.v20.i26.8505 25024606 PMC4093701

[235] Juan W Zhen H Yan-Ying F Hui-Xian Y Tao Z Pei-Fen G A comparative study of two tube feeding methods in patients with dysphagia after stroke: a randomized controlled trial. J Stroke Cerebrovasc Dis. (2020) 29(3):104602. 10.1016/j.jstrokecerebrovasdis.2019.104602 31924485

[236] Kim G Baek S Park HW Kang EK Lee G. Effect of nasogastric tube on aspiration risk: results from 147 patients with dysphagia and literature review. Dysphagia. (2018) 33:731–8. 10.1007/s00455-018-9894-7 29619559

[237] Maeda K Akagi J. Oral care may reduce pneumonia in the tube-fed elderly: a preliminary study. Dysphagia. (2014) 29(5):616–21. 10.1007/s00455-014-9553-6 25034303

[238] Sifuentes AM Lapane KL. Oral health in nursing homes: what we know and what we need to know. J Nurs Home Res Sci. (2020) 6(1):1–5. 10.14283/Jnhrs.2020.1 32524062 PMC7286629

[239] Adachi M Ishihara K Abe S Okuda K. Professional oral health care by dental hygienists reduced respiratory infections in elderly persons requiring nursing care. Int J Dent Hyg. (2007) 5(2):69–74. 10.1111/j.1601-5037.2007.00233.x 17461957

[240] Volk L Spock M Sloane PD Zimmerman S. Improving evidence-based oral health of nursing home residents through coaching by dental hygienists. J Am Med Dir Assoc. (2020) 21(2):281–3. 10.1016/j.jamda.2019.09.022 31780411

[241] Teusner DN Amarasena N Satur J Chrisopoulos S Brennan DS. Applied scope of practice of oral health therapists, dental hygienists and dental therapists. Aust Dent J. (2016) 61(3):342–9. 10.1111/adj.12381 26465634

[242] Ho SY Walsh LJ Pradhan A Yang J Lopez Silva CP. Perspectives of oral health therapists on the barriers to oral care provision in nursing homes in Singapore: a qualitative analysis. Spec Care Dentist. (2024) 44(1):157–65. 10.1111/scd.12833 36752197

[243] Ishikawa A Yoneyama T Hirota K Miyake Y Miyatake K. Professional oral health care reduces the number of oropharyngeal bacteria. J Dent Res. (2008) 87(6):594–8. 10.1177/154405910808700602 18502972

[244] Adachi M Ishihara K Abe S Okuda K Ishikawa T. Effect of professional oral health care on the elderly living in nursing homes. Oral Surg Oral Med Oral Pathol Oral Radiol Endod. (2002) 94(2):191–5. 10.1067/moe.2002.123493 12221387

[245] Sjögren P Wårdh I Zimmerman M Almståhl A Wikström M. Oral care and mortality in older adults with pneumonia in hospitals or nursing homes: systematic review and meta-analysis. J Am Geriatr Soc. (2016) 64(10):2109–15. 10.1111/jgs.14260 27590446

[246] Yoneyama T Yoshida M Ohrui T Mukaiyama H Okamoto H Hoshiba K Oral care reduces pneumonia in older patients in nursing homes. J Am Geriatr Soc. (2002) 50(3):430–3. 10.1046/j.1532-5415.2002.50106.x 11943036

[247] Nakashima S Miyamoto A Takahashi Y Nakahama H Moriguchi S Murase K Mendelson’s syndrome complicated by bacterial aspiration pneumonia triggered by right putamen bleeding: a case report. Resp Med Case Rep. (2021) 33:101466. 10.1016/j.rmcr.2021.101466 PMC8349091 34401302

[248] Lascarrou JB Lissonde F Le Thuaut A Bachoumas K Colin G Lagarrigue MH Antibiotic therapy in comatose mechanically ventilated patients following aspiration: differentiating pneumonia from pneumonitis. Crit Care Med. (2017) 45(8):1268–75. 10.1097/CCM.0000000000002525 28594680

[249] Elsherbiny DH Abo-Shehata ME Elgamal EA Ahmed MA Elgamal MM El-Sayed MA Role of bronchoalveolar lavage in differentiation between bacterial aspiration pneumonia and gastric aspiration pneumonitis. Egypt J Chest Dis Tubercul. (2023) 72(2):160–6. 10.4103/ecdt.ecdt_16_20

[250] Zhu L Hao Y Li W Shi B Dong H Gao P. Significance of pleural effusion detected by metagenomic next-generation sequencing in the diagnosis of aspiration pneumonia. Front Cell Infect Microbiol. (2022) 12:1887. 10.3389/fcimb.2022.992352 PMC9808782 36605125

[251] Klompas M Speck K Howell MD Greene LR Berenholtz SM. Reappraisal of routine oral care with chlorhexidine gluconate for patients receiving mechanical ventilation: systematic review and meta-analysis. JAMA Intern Med. (2014) 174(5):751–61. 10.1001/jamainternmed.2014.359 24663255

